# BrmiR828 Targets *BrPAP1*, *BrMYB82*, and *BrTAS4* Involved in the Light Induced Anthocyanin Biosynthetic Pathway in *Brassica rapa*

**DOI:** 10.3390/ijms21124326

**Published:** 2020-06-17

**Authors:** Bo Zhou, Jingtong Leng, Yanyun Ma, Pengzhen Fan, Yuhua Li, Haifang Yan, Qijiang Xu

**Affiliations:** 1 Key Laboratory of Saline-alkali Vegetation Ecology Restoration (Northeast Forestry University), Ministry of Education, Harbin 150040, China; lyhshen@126.com (Y.L.); yanhaifang224@126.com (H.Y.); 2 College of Life Science, Northeast Forestry University, Harbin 150040, China; lengjt558@163.com (J.L.); myy060528@163.com (Y.M.); fpzvictory@163.com (P.F.)

**Keywords:** anthocyanin biosynthesis, *Brassica rapa*, gene expression, miR828, regulation

## Abstract

Comprehensive research in various plants shows that the metabolic pathway of anthocyanin biosynthesis is affected by environmental factors and regulated by microRNAs through post-transcriptional regulation. In seedlings of *Brassica rapa* Tsuda, the accumulation of anthocyanin is induced by light. However, the roles of BrmiR828 in the light-induced synthesis of anthocyanin in *Brassica rapa* remain to be explored. Here, a primary transcript of BrmiR828 was identified to be located on the chromosomes of the A03 sub-genome. Five candidate *MYB* family genes were predicted as targets of BrmiR828 in the database of *Brassica rapa* (BRAD, V1.1) by using psRNATarget. The transcript abundance of mature BrmiR828 was reduced in seedlings of *Brassica rapa* Tsuda under blue light irradiation comparing with dark treatment. However, Real-time PCR showed the transcript level of the five candidate targets, Bra004162, Bra022602, Bra001917, Bra029113, and Bra039763 was up-regulated when the seedlings exposed to blue or UV-A light. Trans-acting siRNA gene 4 (BrTAS4) was also identified to have a higher transcript level under blue and UV-A light irradiation than that in dark treatment. RNA ligase mediated 5′amplification of cDNA ends (RLM-5′ RACE) showed that BrmiR828 can splice the mRNA of Bra039763, Bra022602, and BrTAS4 on binding sites. Phylogenetic analysis of candidate BrMYBs targets along with MYBs from *Arabidopsis thaliana* showed that Bra039763, Bra004162, Bra001917, Bra029113, and Bra022602 are classified to the same group with AtMYB75, AtMYB114, AtMYB90, AtMYB113, and AtMYB82 which are involved in the anthocyanin biosynthetic pathway. As a result, light-induced down-regulation of BrmiR828 can target *BrTAS4*, *BrPAP1* (Bra039763), *MYB82* (Bra022602) to negatively regulate their transcript levels leading to the accumulation of MYB transcription factors that positively regulate anthocyanin biosynthesis in light-exposed seedlings of *Brassica rapa*.

## 1. Introduction

Anthocyanins are widely distributed in the plant kingdom and affect the color of fruits and vegetables [1,2]. They take important roles in protecting against UV radiation, attracting insects for pollination, pathogen resistance, and drought tolerance [3,4,5]. Also, anthocyanins have been demonstrated to possess antioxidative activity and have potential benefits for human health [6,7]. The pathway of anthocyanin biosynthesis has been clarified and most of the genes involved in anthocyanin biosynthesis and regulation have been identified in model plants [8]. However, the post-transcriptional regulation especially miRNAs involved in the molecular regulation of anthocyanin biosynthesis is still an important research objective.

Light is an important environmental signal to control metabolic processes during plant growth and development [9,10]. Light signaling components such as PIF3, CYR1, UVR8, and HY5 have been demonstrated to be key light signal transduction factors involved in anthocyanin biosynthesis under different light conditions [11,12,13,14]. Blue and UV-A light have also been reported to induce the expression of genes involved in anthocyanin biosynthesis in *Brassica rapa* [15,16], *Litchi chinensis* [17], *Lactuca sativa* [18], *Arabidopsis* [12], and *Pyrus* [19]. miRNAs negatively regulate the expression of genes involved in light-regulated processes [20,21]. Analysis of UV-B regulated miRNAs showed stress-responsive cis-elements located in promoters of *MIR* genes are speculated to be involved in responses to abiotic and biotic stresses [22,23,24]. Also, HY5 was shown to bind the promoters of *MIR156D*, *MIR402*, *MIR408*, and *MIR858A* and regulate their expression in *Arabidopsis* [25]. Analysis of the upstream regions of UV-B regulated *MIRNA* genes showed several light-related motifs, such as G box, GT-1 site, I-box, CCAAT-box, and GATA-box exist in regulatory regions and consistent with the effect of light on these genes [22,26]. Then light strongly affects the transcription level of *MIRs* and further research needs to be conducted to explore the roles of miRNAs and their target genes in light-induced anthocyanin biosynthesis process.

Transcription factors take important roles in regulating the transcription of genes involved in anthocyanin biosynthesis. R2R3 MYB, basic helix-loop-helix (bHLH) and WD repeat (WDR) transcription factors can form a trimeric MYB -bHLH- WD40 (MBW) complex to activate the expression of late pathway genes including dihydroflavonol reductase (DFR), anthocyanidin synthase (ANS), and flavonol-3-glucosyltransferase (3GT) [27,28,29]. In *Arabidopsis*, four genes *PAP1/MYB75*, *PAP2/MYB90*, *MYB113*, and *MYB114* encoded R2R3-MYB proteins with high sequence similarities have been identified to positively regulate anthocyanin biosynthesis in vegetative tissues [30,31]. Also, GL3, EGL3, and TT8, the members of the bHLH transcription factor family have been proved to regulate anthocyanin biosynthesis [32,33,34]. Moreover, TTG1, the member of the WD40 protein determines the regulation of anthocyanin biosynthesis in *Arabidopsis* [35]. However, recent research shows that MYB-LIKE2 (MYBL2) and Squamosa promoter binding protein-like 9 (SPL9) negatively regulate anthocyanin biosynthesis through interfering with the formation of the MBW complex [36,37]. Then the quantitative competition between positive MBW regulators and negative regulators MYBL2/SPL9 can determine the activation/repression of the expression of anthocyanin biosynthetic pathway genes [38].

In plants, miRNAs play critical regulatory roles by negatively regulating the expression of transcription factors and metabolism-related proteins that modulate growth, development, and abiotic stress responses at the post-transcriptional level [36,39,40,41]. Recently, miRNAs were reported to be involved in anthocyanin biosynthesis in *Arabidopsis* [25,36,42,43]. MiR156 has been demonstrated to negatively regulate the transcription of *SPL9*, the protein product of which can compete with bHLH proteins to interact with PAP1 (Production of Anthocyanin Pigment 1) and positively regulate the accumulation of anthocyanins in the inflorescent stem [36]. MiR408, a positive regulator of photomorphogenesis, can lead to anthocyanin accumulation in seedlings [25,42]. MiR858a has also been demonstrated to enhance anthocyanin biosynthesis in seedlings by inhibiting the expression of *MYBL2* through translational repression [43]. However, in *Solanum lycopersicum*, miR858 negatively regulates the anthocyanin pathway by modulating *SlMYB48-like* and *SlMYB7-like* transcripts [44]. Moreover, anthocyanin biosynthesis is also reported to be negatively regulated by miR828 or TAS D4(−) (previously TAS4-siRNA81(−)) in *Arabidopsis* [45,46,47]. Then miRNAs seem to have dual regulating roles in anthocyanin biosynthesis in different plants.

MiR828 was initially identified in *Arabidopsis* and functioned as the negative regulator of anthocyanin biosynthesis [45,47]. MiR828 mediates the cleavage of TAS4 and initiates the production of TAS D4(−) which is predicted to target *MYB113*, *MYB75*, and *MYB90* genes that are involved in anthocyanin biosynthesis [47]. MiR828 has also been predicted to directly target *MYB113* [46,48]. Over-expression of miR828 results in reduced expression of *MYB113*, *MYB75*, *MYB82*, and *MYB90* and anthocyanin accumulation in *Arabidopsis* [45]. In apple (*Malus × domestica*), miR828, miR858, and TAS4 D4(−) has also been identified in high-throughput sequencing and was found to target 81 *MYB* genes [39]. Our previous research has characterized the miRNAs in response to blue/UV-A light in *Brassica rapa* [49], but the function of miR828 involved in light-induced anthocyanin biosynthesis in *Brassica rapa* remains to be elucidated.

Turnip (*Brassica rapa* subsp. *Rapa*; *Brassicaceae*) is an important and popular cruciferous root vegetable. The seedlings of turnip cv. Tsuda can accumulate anthocyanin in hypocotyls after exposure to sunlight, blue light, and UV-A light [16,49]. Among UV-B, UV-A, blue, red, and far-red lights, UV-A specific induces anthocyanin biosynthesis in the hypocotyls of turnip [50]. This study aimed to determine the regulation of BrmiR828 and its targets in blue light and UV-A induced anthocyanin biosynthesis of *Brassica rapa*. Based on the data, we demonstrate that BrmiR828 targets the transcripts of *BrPAP1*, *BrMYB82*, and *BrTAS4* by RLM-5′ RACE. The transcript abundance of *BrMIR828* decreased while the expression of its targets *BrPAP1*, *BrPAP2*, *BrMYB82*, and *BrTAS4* greatly increased after blue light induction, indicating that BrmiR828 negatively controls light-induced anthocyanin biosynthetic pathway by repressing the expression of *BrPAP1*, *BrPAP2*, *BrMYB82,* and *BrTAS4* genes in *Brassica rapa*.

## 2. Results

### 2.1. Light-Induced Anthocyanin Biosynthesis in Outer Epidermal Cells of Hypocotyls

**Figure 1:**
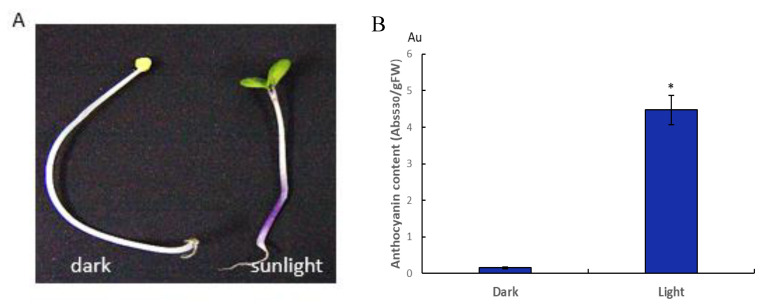
The phenotype of *Brassica rapa* subsp. *rapa* cv. Tsuda seedling and anthocyanin content after dark and sunlight treatment. (**A**) Seedlings were exposed to sunlight or darkness. (**B**) Anthocyanin was extracted from fresh whole seedlings, and the concentration was determined using absorbance at 530 nm per gram of fresh mass. Vertical bars indicate ± SE of replicate assays (*n* = 8). The statistically significant difference between light treatment and dark is shown (*t*-test, * *p* < 0.05).

**Figure 2:**
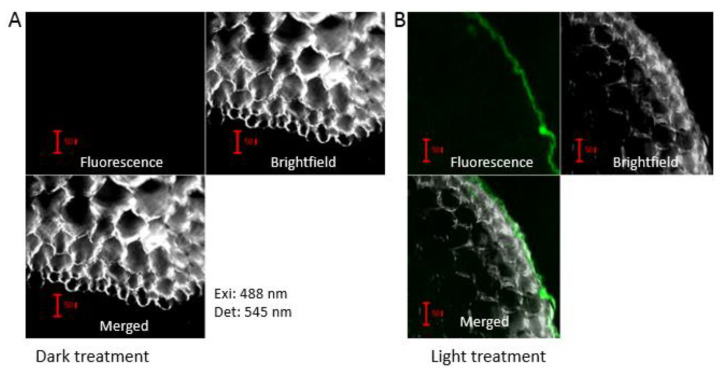
Anthocyanin distribution in the cross-section of the hypocotyl. Samples were prepared through the hand-sliced method and observed using a confocal microscope. Anthocyanin detection using 488 nm excitation and 545 nm emission. Samples of dark (**A**) and light (**B**) treatment viewed with anthocyanin detection field, bright field, and merged images. The scale bars in the figure represent 50 µm. The green color is indicative of anthocyanin presence.

When the seedlings of *Brassica rapa* were exposed to sunlight, pigments could accumulate in the hypocotyls of seedlings and showed a light-induced photomorphogenic phenotype with de-etiolation, phototropism, cotyledon expansion, chlorophyll synthesis, and short hypocotyls. Also, the content of anthocyanin in light-induced seedlings is much higher than that in dark treated seedlings (Figure 1). To clarify the distribution of anthocyanin in epidermal cells of hypocotyls, a hypocotyl cross-section was cut and examined using a confocal microscope. The result showed that anthocyanins synthesize in the outermost layer cells of the light treated hypocotyls and no anthocyanins accumulate in outer epidermal cells of dark treated hypocotyls (Figure 2).

### 2.2. Sequence Analysis of Primary miR828 Transcripts and Targets Identification of BrmiR828 in Brassica rapa

**Figure 3:**
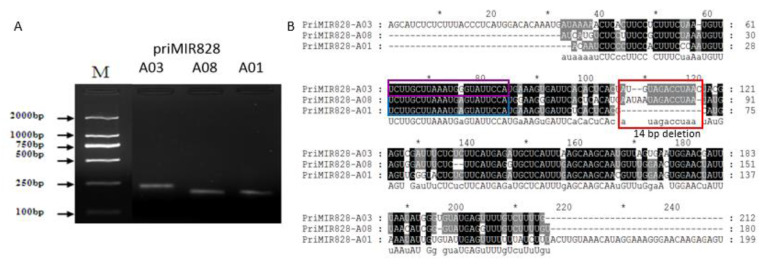
Three candidate primary miR828 transcripts were identified by qRT-PCR and sequences alignment showed they are respectively located in *B. rapa* chromosome A03, A08, and A01. (**A**) Electrophoresis of PCR product on agarose gel. (**B**) The sequences of these primary miR828 transcripts are highly similar except that primiR828-A01 has a 14 bp sequence deletion. The sequences in blue box and purple box respectively represent the region of miR828a and miR828b.

Through high throughput sequencing, the sequence of BrmiR828 was obtained in *Brassica rapa* [49]. To further study the regulatory roles of BrmiR828 in the anthocyanin biosynthesis pathway, primary candidate miR828 transcripts were identified and the target genes of miR828 were also predicted for analysis. Three candidate primary miR828 transcripts were identified by homology cloning and sequences alignment showed they are respectively located in *B. rapa* chromosome A03, A08, and A01. The sequences of these primary miR828 transcripts are highly similar except that primiR828-A01 has a 13–14 bp sequence deletion compared with primiR828-A01 and primiR828-A08 (Figure 3) and they are all predicted to have a stem-loop structure by Mfold [51] (Figure S1). According to the predicted stem-loop structure, there should be two kinds of mature miR828 sequences, BrmiR828a and BrmiR828b. BrmiR828a (from A01 and A08) is identical with AtmiR828 from *Arabidopsis*, and BrmiR828b (from A03) has only one base different with either AtmiR828 from *Arabidopsis* or SlmiR828 from *Solanum lycopersicum* (Figure S2). Only BrmiR828b was identified by sequencing and the obtained mature BrmiR828 might derive from the strand of the pre-miRNA (from A03) stem which was processed in the nucleus by RNase complex. Then the mature miR828 from *Brassica rapa* was deduced to have the same function as miR828 from *Arabidopsis* or *Solanum lycopersicum*. The prediction of miR828 targets showed five candidate MYB transcripts and one protein kinase family gene are directly bound by miR828 and regulated by cleavage using psRNATarget [52] (Table 1). Moreover, the transcript of a non-coding RNA, *Trans*-acting siRNA gene 4 (*TAS4*) cloned from *Brassica rapa* was also predicted to be cleaved by miR828.

### 2.3. Phylogenetic Analysis of the Candidate Target MYB Genes in Brassica rapa with MYB Gene Family in Arabidopsis

**Figure 4:**
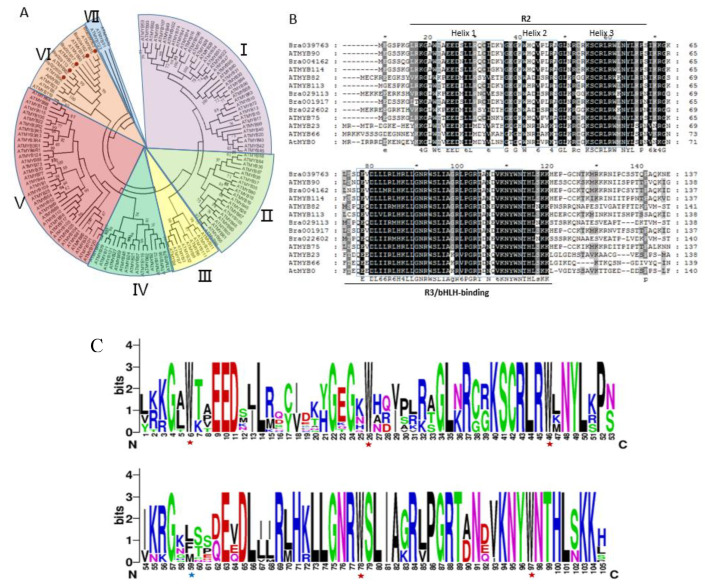
Phylogenetic analysis of candidate BrMYBs and multiple sequence alignment of R2R3 MYB family in *Brassica rapa* and AtMYBs in *Arabidopsis*. (**A**) The phylogenetic tree was constructed by using the Neighbor-Joining method [58] with 1000 bootstrap replicates [59]. Branches corresponding to partitions reproduced in less than 40% bootstrap replicates are collapsed. The evolutionary distances were computed using the Poisson correction method [60] and were conducted in MEGA7 [61] I to VII represents the MYB proteins divided subgroup I to subgroup VII, separately. (**B**) Protein sequences of subgroup VI were aligned by using Clustal X ver. 2 [62] with default settings, and the conserved amino acids were shaded by using GeneDoc (2.6) [63]. The R2, R3/bHLH-binding domains are marked with black bars and three α-helices of both R2 and R3 domains are indicated by square frame [64]. (**C**) The R2R3-domain sequences from subgroup VI were aligned and the sequence conservation at a particular position expressed as a stack of letters using the WebLogo program [65]. The overall height of each stack indicates the sequence conservation at that position (measured in bits), whereas the height of symbols within the stack reflects the relative frequency of the corresponding amino acid. The conserved tryptophan residues (Trp, W) in the MYB domain are marked with red asterisks. The replaced residues in the R3 repeat are shown by blue asterisks.

To investigate the phylogenic relationship between the target *MYB* genes of miR828 in *Brassica rapa* and *MYB* family genes in *Arabidopsis*, an unrooted phylogenetic tree was constructed using Neighbor-Joining method, based on the alignment amino acid sequences of candidate BrMYBs with 132 AtMYBs (Table S3). The phylogenic tree showed that all the MYB family proteins were divided into seven subgroups and the target *MYB* genes of miR828 were all clustered into subgroup VI (Figure 4A). Moreover, in subgroup VI, five *Arabidopsis* MYB genes encoding AtMYB75, AtMYB113, AtMYB114, AtMYB90, and AtMYB82 have been identified to be involved in anthocyanin biosynthesis [38,45,53]. Phylogenetic analysis indicates that the BrMYBs clustered with already characterized AtMYBs have similar functions. Multiple sequence alignment of MYBs in subgroup VI showed two DNA-binding domains, R2 and R3 are conserved and each domain has three α-helices which are reported to be critical for contacting with DNA (Figure 4B) [54,55]. Furthermore, a conserved motif DLX2RX3LX6LX3R in the first two α-helices of the R3 domain was also reported to be involved in MYB-bHLH interactions (Figure 4B) [54,55,56,57]. Three of five BrMYBs (BrPAP1 (Bra039763), BrPAP2 (Bra004162 and Bra001917)) showed similarity to AtMYB75, AtMYB113, AtMYB114, and AtMYB90. Bra039763 had a substitution at Arg63 (R) by Lys (K). The other two BrMYBs (Bra022602, Bra029113) were similar to AtMYB82 and putative BrMYB82 (Bra022602) had a substitution at Ser71 (S) by Thr (T) in the region between R2 and R3 domains (Figure 4C). The frequency analysis of the most prevalent amino acids at each position within each repeat of the R2R3-MYB domains showed highly conserved triplet tryptophan (Trp, W) residues are located at positions 6, 26, and 46 of the R2 domain and 59, 78, and 97 of the R3 domain in MYBs of *Brassica rapa*. In the R3 repeat, the first tryptophan (Trp59) residue was also usually replaced by isoleucine or phenylalanine (Ile, I or Phe, F) (Figure 4C).

### 2.4. Light-Dependent Expression Analysis of BrMIR828 and Its Candidate Target Genes under Blue Light and UV-A

**Figure 5:**
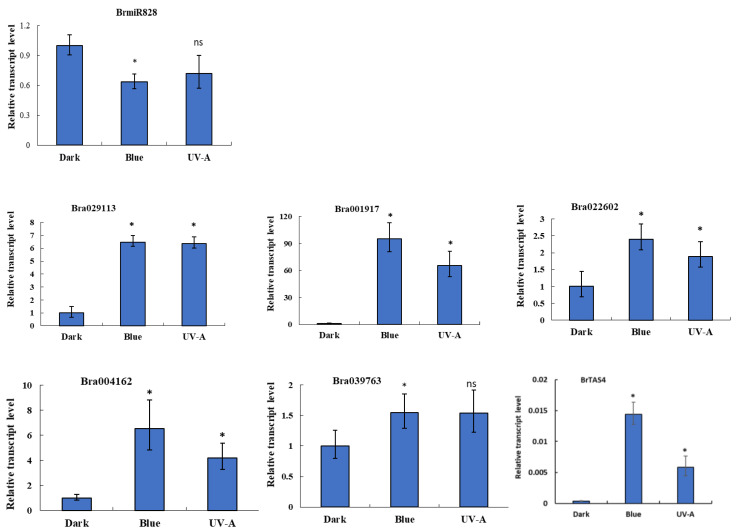
Relative expression of BrmiR828 and its candidate targets in seedlings of *Brassica rapa* with dark, blue, or UV-A light treatment was detected by qRT-PCR and calculated with 2^−ΔΔCT^. The ubiquitin (*UBQ*) gene served as the internal control to correct for template quantity. Each bar indicates the mean ± SE of three biological replicates assays after correcting for template quantity relative to the *UBQ* gene. Statistically significant differences between blue light treatment and dark, UV-A treatment and dark are shown (*t*-test, * *p* < 0.05; ns, not significant difference, *df* = 3)

To explore the expression character of *BrMIR828* and its candidate target genes responsive to dark, blue, and UV-A light, their transcription level was detected by real-time PCR. The results showed that a significant change in abundance of BrmiR828 after blue light treatment compared with the dark treatment (*p* < 0.05) (two-tailed t-test, the *p*-value is 0.0267, *t* = 4.303, *df* = 3), but under UV-A light treatment compared with the dark treatment the abundance did not differ significantly (*p* < 0.05) (*p*-value is 0.0602, *t* = 4.303, *df* = 3) (Figure 5). In the candidate targets of BrmiR828, the transcript level of Bra039763 had no obvious difference (*p* < 0.05) (*p*-value is 0.0660, *t* = 4.303, *df* = 3) under UV-A light compared with dark treatment, and the others showed both blue and UV-A light-induced expression. The mRNA level of predicted targets *Bra001917*, *Bra029113*, and *Bra004162* induced by blue light and UV-A was over five times as much as that in the dark treatment. However, the transcript level of *Bra039763* was only about a 1.5-fold change in blue and UV-A light compared with that in dark treatment. The transcription level of *BrTAS4* was also greatly increased after blue and UV-A light induction (Figure 5). The reversed expression patterns of target genes and *BrMIR828* suggest post-transcription level regulation occurs between them.

### 2.5. BrmiR828 Guided the Cleavage of BrPAP1, BrMYB82 and BrTAS4 to Inhibit Their Expression

**Figure 6:**
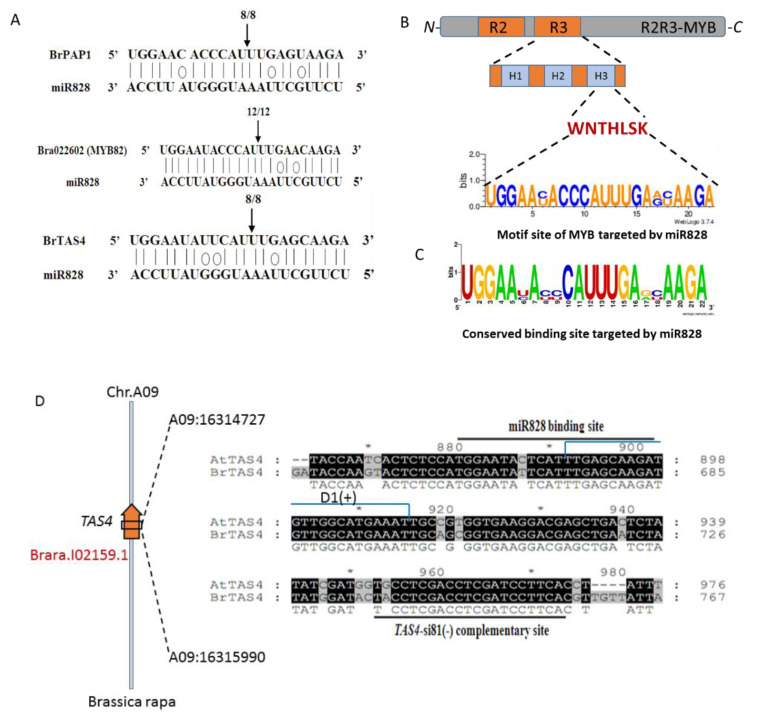
Sites of BrmiR828-mediated cleavage on target *BrPAP1* (Bra039763), *BrMYB82* (Bra022602) mRNAs and *BrTAS4* and chromosome location and sequence alignment of TAS4 orthologs in *Brassica rapa* and *Arabidopsis thaliana*. (**A**) Positions of dominant 5′-RACE products of *BrPAP1*, *BrMYB82,* and *BrTAS4* are indicated by a vertical arrow in the binding region. (**B**) Schematic diagram of the motif site of *BrMYB* targeted by BrmiR828, H1-3 are helices. (**C**) RLM 5′-RACE verified conserved binding site targeted by BrmiR828. (**D**) The sequences designated by dark line shows the binding site of miR828 and the complementary site of TAS4-siR81(−) [46]. The first siRNA, TAS4 D1(+) originated from the splicing site of BrmiR828 and TAS4.

To confirm the regulation between BrmiR828 and their candidate targets, RLM-5′ RACE was used to detect the binding site and cleavage site. Sequencing of the 5′RACE PCR clones revealed that the miR828-guided cleavage of target *BrPAP1* mRNA (Bra039763), *BrMYB82* mRNA (Bra022602), and *BrTAS4* mRNA occur as expected. Also, the cleavage sites were identified between 10 and 11 bases from the 5′ ends of the binding region in the BrmiR828 sequence (Figure 6A) and the motif site of *MYB* was targeted by miR828 coding for helix 3 of specific R3 domain (Figure 6B). Then, a conserved binding site targeted by BrmiR828 verified through RLM 5′-RACE can be concluded (Figure 6C). Unfortunately, we have not detected the cleavage sites of other candidate targets such as Bra001917, Bra004162, and Bra029113. Further analysis showed *BrTAS4* is located on chromosome 9 of *Brassica rapa* (Figure 6D). After cleavage by BrmiR828, small RNAs (TAS4 siRNAs) were identified (Table 2) by high throughput sequencing [49] and one of the TAS4 siRNAs or siRNA TAS D4(−) was also predicted to target Bra039763 (*BrPAP1*), Bra004162 (*BrPAP2*), and Bra001917 (*BrPAP2*) (Figure S3).

## 3. Discussion

In this study, the role of miR828 involved in light-induced anthocyanin biosynthesis was investigated. Numerous studies indicate that various abiotic factors such as light [66], temperature [67], nitrogen and phosphate deficiencies [68], and sucrose [69] induce anthocyanin biosynthesis. Light is one of the most important environmental factors regulating the biosynthesis of anthocyanins. For example, blue and UV-B light have also been reported to mediate miRNAs that regulate genes in maize and *Arabidopsis* [23,70]. Anthocyanins are often presented in the epidermis of flowers and fruits to attract pollinators and seed dispersers [71]. In the hypocotyl of *Brassica rapa* seedlings, the pigment also accumulated in the outer layer cells of the epidermis after light induction (Figure 2). The anthocyanin accumulation is related to light-mediated photomorphogenic responses through photoreceptors and they might take resistance roles to UV damage.

miRNAs are known to be involved in various multiple biological processes such as development, primary and secondary metabolism, and stress responses [36,39]. In *Arabidopsis*, miR828 acts as a negative regulator and inhibits anthocyanin biosynthesis at different developmental stages [45]. In tomato, anthocyanin content was also greatly reduced in overexpressed AtmiR828 transgenic plants [72]. AtmiR828 encoded by a single locus lies in chromosome 4 (AT4G27765) which can form a stem-loop hairpin. By high throughput sequencing [49], a 22 nt long mature miR828 was also identified with almost the same sequences to AtmiR828 in *Brassica rapa*. *MIR* genes encoding identical mature miRNAs can differ in gene structure and regulatory sequences and express in response to various environmental stimuli [73]. It is necessary to identify the primary transcripts and the locus of *miR828* in *Brassica rapa*. Although, three candidate *MIR* loci in chromosome A03, A08, A01 were obtained (Figure 3B) and predicted to form a stem-loop structure (Figure S1), only the mature miR828 originated from chromosome A03 was detected by sequencing [49]. The candidate mature miRNA from chromosome A08 and A01 still need experimental verification. Also, the candidate loci in chromosome A01 and A08 might be pseudo *MIR* gene. This might be due to several chromosome rearrangements events, leading to the evolution of *Brassica rapa* (AA, 2n = 20) [74]. Then the mature miR828 originated from chromosome A03 was used to predict the targets of miR828.

Among the predicted target genes of miR828, five genes were annotated to encode MYB protein. Phylogenetic analysis showed that candidate target MYB genes in *Brassica rapa* are clustered with AtMYB75, AtMYB113, AtMYB114, AtMYB90, and AtMYB82 in *Arabidopsis* (Figure 4A). AtMYB75 also known as Production of Anthocyanin Pigment 1 (PAP1) have been identified to control anthocyanin biosynthesis in vegetative tissues [75]. Overexpression of *AtMYB90* (*PAP2*), AtMYB113, and *AtMYB114* can also lead to anthocyanin accumulation in leaves and seedlings of *Arabidopsis* [33,75]. In addition, MYB82 was demonstrated to function in the regulation of trichome development [76] and might also take a potential role in the regulation of anthocyanin biosynthesis [45]. The results of the phylogenetic analysis indicate that the candidate BrPAP1 (Bra039763), BrPAP2 (Bra001917 and Bra004162), and BrMYB82 (Bra029113) might also function as a regulator of anthocyanin biosynthesis in *Brassica rapa*. Among these BrMYBs, each of the R2 and R3 domains contains three helices (Figure 4B), and helices 1 and 2 of the R3 domain can form a bHLH binding domain to interact with bHLH proteins [57]. The third helix is relatively conserved and important for DNA binding activity and the residues within the region between R2 and R3 domains are related to protein-DNA binding ability [77,78]. The results indicate that these conserved MYB domains can interact with bHLH protein together with WD repeat transcription factors to form MBW complex to activate the transcription of anthocyanin biosynthetic pathway genes including *DFR*, *ANS*, and *3-GT* just like AtMYB75 in *Arabidopsis* [27,38,79].

Co-expression analysis of miRNAs and target genes is essential to understand the regulation relationship between them. A negative correlation between BrmiR828 and its predicted targets Bra022602 (*MYB82*), Bra029113 (*MYB82*), Bra001917 (*PAP2*), Bra004162 (*PAP2*), and Bra039763 (*PAP1*) was detected in dark and blue light treated seedlings by qRT-PCR analysis (Figure 5) and suggested that BrmiR828 directly mediate their cleavages in mRNA level. The mechanism is different from miR858a regulating MYBL2 through repressing translation [43]. Although the expression of BrmiR828 and Bra039763 did not show obvious differences under UV-A light and dark treatment, their regulatory roles in light-induced anthocyanin biosynthesis could not be ignored. The final expression level of the miRNA and its target gene which may not be correlated has been reported to be related to spatial restriction, mutual exclusion, dampening, and temporal regulation [80]. We also found that the abundance of BrmiR828 was also lower than that of other miRNAs such as miR156, miR159 detected by high throughput sequencing, and qRT-PCR [49]. Additionally, miR828 was also detected with low abundance in *Malus × domestica* [39], *Arabidopsis* [45], *Prunus persica* [81], and *Vitis* [82], and the functional role of miR828 has been proved to regulate the development of *Arabidopsis* trichome and cotton fiber [83], the anthocyanin biosynthesis of *Vitis* [82] and *Arabidopsis* [45]. A *Brassica rapa TAS4* ortholog, defined as *BrTAS4*, was also identified to be a candidate target of miR828 and the sequence of miR828 binding site is similar to that of TAS4 in *Arabidopsis* except for a substitution of C by T base (Figure 6D). Only 24 bp length siRNAs from BrTAS4 were identified in *Brassica rapa* [49] and in *Arabidopsis*, the siRNAs were also mostly 24 mers [48]. Moreover, the expression of BrTAS4 was increased with blue and UV-A light treatment and showed a negative correlation with the expression of BrmiR828 under dark and blue light treatment. TAS4-siR81(−), one of the small RNAs originated from TAS4, was shown to target *PAP1*, *PAP2*, and *MYB113* [46]. Furthermore, miR828 and TAS4 are also positively regulated by PAP1, and a negative feedback loop regulates the expression of *PAP1* and its homologs [47]. These results have shown that a potential autoregulatory mechanism of the expression of anthocyanin pathway genes through sRNAs including miR828, TAS4-siR81(−), and miR156/157 [38,49].

Through RLM-5′ RACE, the cleavage site between miRNA and its target can be identified experimentally [84]. In the present study, we have obtained the binding cleavage site of BrmiR828 in *BrPAP1* (Bra039763), *BrMYB82* (Bra022602), and *BrTAS4* and confirmed the direct cleavage regulation of miR828 to them in vivo. However, the cleavage site of other candidate targets (Bra001917, Bra004162, and Bra029113) have not been obtained in our research. Extensive base-pairing between the miRNA and the mRNA was not always sufficient to induce cleavage, and there can be additional requirements for a RNA-induced silencing complex (RISC) complex to catalyze endonucleolytic cleavage [85]. Also, these candidate *MYBs* might be targeted by other miRNAs/siRNAs, such as miR159, miR858, and tasiRNAs which have been reported to regulate *MYBs* in *Malus × domestica* [39]. To elucidate the regulation of miR828 and its targets, comparing the phenotypic impact of the expression of the miRNA-resistant target to its wild-type counterpart can be adopted [86]. In *Arabidopsis*, miR828 targeting *AtMYB75*, *AtMYB113*, *AtMYB82*, and *TAS4* regulates the expression of genes in the anthocyanin biosynthetic pathway including *DFR* and *LDOX* [45,47]. MiR828 and miR858 have also been proved to regulate MYB114 to accumulate anthocyanin in *Vitis* [82]. Our present results are consistent with these findings and demonstrate that the mechanism of miR828 involved in anthocyanin biosynthesis is also conserved in *Brassica rapa*. Here, we also confirmed that BrmiR828 directly mediates the cleavage of BrMYB82 in *Brassica rapa* by RLM-5′ RACE. In *Arabidopsis*, the elevated level of miR828 inhibits the transcription of MYB82, indicating a potential role of MYB82 in the regulation of anthocyanin biosynthesis [45]. However, direct involvement of MYB82 in anthocyanin biosynthesis has not been demonstrated in any plant species except that MYB82 functions in the regulation of trichome development in *Arabidopsis* [76]. The regulated role of BrMYB82 on anthocyanin biosynthesis might be indirect in the pathway and our previous research showed miR156/157-guided cleavage of target SPL9 and SPL15 [49] also indirectly control anthocyanin accumulation by disrupting the stabilization of the MYB-bHLH-WD40 MBW complex [36]. Then the function for target MYB82 of miR828 in the pathway of anthocyanin biosynthesis needs to be done in the future.

The function of BrmiR828 and its transcription factor targets involved in anthocyanin biosynthesis should be detected through over or knockdown expression of these genes in *Brassica rapa* (turnip) plants. However, the generation of transgenic *Brassica rapa* plants has been reported to be difficult [87]. Although different transformation efficiencies vary among different genotypes and species of *Brassica*, compared with other *Brassica* species, *Brassica rapa* is the most recalcitrant species to transform [88]. Nowadays, the genetic transformation of turnip (*Brassica rapa* subsp. *rapa*) which form tubers has not been overcome. Overexpressing *SlMYB75* (ortholog of *BrPAP1*) has been reported to effectively induce the accumulation of anthocyanin in both vegetative and reproductive organs of tomato [89]. Then the agroinfiltration-mediated transient expression system in tomato can be used to investigate the function of *BrPAP1*. When *BrPAP1* overexpressed in tomato fruits through transient expression, pigment accumulated and the expression levels of *SlCHS*, *SlDFR*, and *SlANS* genes which involved in anthocyanin biosynthesis also increased [90]. The research suggested that *BrPAP1*, the target of BrmiR828, is involved in anthocyanin biosynthesis in *Brassica rapa*. Also, the function of *BrMYB82* and *BrTAS4* needs to be further explored by overexpression or knockdown analysis in the pathway of anthocyanin synthesis and metabolism in the future.

## 4. Materials and Methods

### 4.1. Plant Materials

Based on the previous research, we have known that blue light and UV-A light spectra were effect to accumulate anthocyanin in sunlight [16]. Then seeds of the turnip *Brassica rapa* L. subsp. *rapa* cv. Tsuda were sown in a row on wet filter paper and grown in the dark at 25 °C for 3 days, then irradiated with blue light (470 nm light-emitting diode LED, NSPB5205, Nichia, Tokushima, Japan) at 10 W m-2 or UV-A light (UV-A fluorescent lamp, FL10BLB, Toshiba, Japan, filtered through soda-lime glass plates, peak at 350 nm) at 3 W m-2 for 24 h. For the control group, the seedlings were still grown in the dark. Then light treated and dark-grown seedlings were used as materials for RNA isolation and real-time PCR. For anthocyanin measurements and cross-section analysis, the seedlings grew in sunlight or darkness for four days separately.

### 4.2. Anthocyanin Measurements

Approximate 0.1 g of four-day-old seedlings was weighed and ground in liquid nitrogen, and the pigments were extracted in 1 mL of 1% HCl in methanol (*v/v*) overnight at 4 °C with continuous shaking. For removing chlorophylls, 1 mL chloroform and 0.5 mL of water were added into the extracts and mixed to centrifuge at 15,000× *g* for 2 min. The top layer was collected and the quantity of anthocyanins was determined by spectrophotometric measurement of the aqueous phase (A530-A657) (Varian, Cary 50 Bio, Melbourne, Australia), and normalized to the total fresh weight of tissue used in each sample. (Modified after Martin et al. 2002). Eight biological replicates were prepared for light and dark treatment in anthocyanin analysis. Anthocyanin was determined by calculating the ratio of Abs530 to gram fresh mass [49].

### 4.3. Fluorescent Microscopic Observation

The hypocotyls of seedlings grown in dark and sunlight were used to obtain tissue slices. The tissue sections were made by a hand-sliced method, and put on a glass slide covered with a cover slip. Then the crosscut slices of hypocotyls were observed with a confocal laser scanning microscope (510 META, from Carl Zeiss, Jena, Germany). The argon laser with output at 488 nm unit was set for anthocyanin excitation and an emission filter 530 nm was used to detect the emission spectrum of fluorescence.

### 4.4. Prediction Potential Target Genes of BrmiR828

The potential targets of BrmiR828 were predicted by the psRNA Target program [52] using default parameters except Expectation set 3.0. *Brassica rapa*, de novo scaffolds assembly v1.1 (2011–08–30) cds were used as the cDNA library for the target search. The functional annotations of these target genes were then analyzed using the BLASTn search in the NCBI database.

### 4.5. WebLogo and Phylogenetic Analysis

The distribution of conserved R2R3-type MYB amino acid residues at the corresponding positions in domain profiles were generated using the Weblogo program with default parameters (http://weblogo.berkeley.edu/logo.cgi, v2.8.2) [65]. The alignments of full-length amino acid sequences of MYBs from *Arabidopsis* and *Brassica rapa* (Table S3) were aligned using ClustalX 2.1 [62] with default settings. Then the phylogenetic tree was constructed based on the alignments using MEGA 7.0 [61] with the Neighbor-joining method [58]. The parameters used in the tree construction were the Poisson model [60] plus uniform rates and 1000 bootstraps [59].

### 4.6. RNA Extraction and Quality Detection

Six seedlings in each sample with UV-A, blue light, and dark treatment were collected and isolated total RNA using TRNzol-A+ reagent according to the manufacturer’s instructions (TIANGEN Biotech, Beijing, China). The concentration and quality of total RNA were detected using 1% agar gel electrophoresis and NanoDrop 2000 Spectrophotometer (Thermo Fisher Scientific, Wilmington, NC, USA).

### 4.7. Quantitative Real-Time PCR

Total RNA of each sample was extracted from six seedlings of *Brassica rapa* under dark, blue light or UV-A light treatment. For cDNA synthesis, 2 µg of total RNA of each sample was reverse transcribed with stem-loop primers for miRNAs and specific reverse primers for the housekeeping and target genes using Reagent Kit with gDNA Eraser (Takara, Dalian, China). All primer sequences are listed in Table S1. The qRT-PCR was performed using the POWER SYBR GREEN PCR Master Mix (Applied Biosystems, Foster City, CA, USA) and ABI 7500 real-time system (Applied Biosystems). Each reaction with a total volume of 20 µL contained 10 µL of PCR Master Mix, 1 µL of the first-strand cDNA as a template and 0.5 µM of each primer. The amplification program consisted of 95 °C for 30 s, followed by 40 cycles of 95 °C for 15 s, 62 °C for 35 s, and 72 °C for 35 s, then added melt gradient-dissociation curves. Three light and dark treatment replicates and three qRT-PCR experimental replicates were performed for each of the detected genes and samples. The fragment of the UBQ gene was amplified as an internal reference control for the normalization of data. A comparative CT (ΔΔCT) method was used for the analysis of the relative amounts of the transcripts [91]. Student’s t-tests were used to analyze the data, and a difference was considered to be statistically significant when *p* < 0.05, the data of three biological replicates was used for statistical analysis.

### 4.8. 5′ RNA Ligase-Mediated RACE (RLM-5′ RACE) PCR

Total RNA (3 µg) from different light-treated seedlings was mixed equally and used for RLM-5′ RACE analysis. The 5′ RACE adapter was directly ligated to mRNA according to the manufacturer’s instructions of the 5′-Full RACE Kit (TaKaRa, Dalian, China) without the alkaline phosphatase and tobacco acid pyrophosphatase treatment. The nested 5′ RACE Outer/Inner primer and two or three gene-specific nested primers were used for the rapid amplification of cDNA ends (Table S2). Then the PCR products were purified and cloned into the pEASY-T5 zero cloning vector (TransGen Biotech, Beijing, China) for sequencing.

## 5. Conclusions

We show here that during anthocyanin accumulating in light-induced seedlings of *Brassica rapa*, the transcript level of *BrPAP1*, *BrMYB82*, and *BrTAS4* is positively related to the content of pigment and *BrPAP1* has been proved to be involved in anthocyanin biosynthetic pathway. BrmiR828 directly splices mRNA of *BrPAP1*, *BrMYB82*, and *BrTAS4* and negatively regulates their expression in seedlings of light treatment. Therefore, BrmiR828 targets *BrPAP1*, *BrMYB82*, and *BrTAS4* involved in light-induced anthocyanin biosynthetic pathway in *Brassica rapa*. The current findings supply a clue to facilitate the production of rich anthocyanin in plants for food with potential benefits to human health.

## Figures and Tables

**Table 1:** The predict targets of miR828 and annotation by psRNATarget using *Brassica rapa* cds library (version 1.1, released on 30 August 2011).

Target Gene ID	Alignment	Target Gene Annotation	Expect ^1^	UPE ^2^
Bra001917	miR828 22 ACCUUAUGGGUAAAUUCGUUCU 1 Target 310 UGGAACACCCAUCUGAGCAAGA 331	ATMYB90/PAP2	2.5	13.531
Bra004162	miR828 22 ACCUUAUGGGUAAAUUCGUUCU 1 Target 310 UGGAACACCCAUUUGAGUAAGA 331	MYB domain protein MYB90/ PAP2	2.0	16.874
Bra004167	miR828 22 ACCUUAUGGGUAAAUUCGUUCU 1 Target 1071 UCGAAAGCCCAUUGAAGUAAGA 1092	protein kinase family protein	3.0	19.602
Bra022602	miR828 22 ACCUUAUGGGUAAAUUCGUUCU 1 Target 322 UGGAAUACCCAUUUGAACAAGA 343	MYB domain protein MYB82	2.0	16.804
Bra029113	miR828 22 ACCUUAUGGGUAAAUUCGUUCU 1 Target 322 UGGAAUACACAUUUGAACAAGA 343	MYB domain protein MYB82	3.0	13.444
Bra039763	miR828 22 ACCUUAUGGGUAAAUUCGUUCU 1 Target 310 UGGAACACCCAUUUGAGUAAGA 331	PAP1	2.0	10.13
BraTAS4	miRNA 22 ACCUUAUGGGUAAAUUCGUUCU 1 Target 663 UGGAAUAUUCAUUUGAGCAAGA 684	Non-coding RNA (*Trans*-acting siRNA gene 4)	1.5	N/A

^1^ Expect value, maximum cutoff of score based on given scoring schema. ^2^ UPE value, Target accessibility, the energy required to unpair target and small RNA.

**Table 2:** The phased tas4 siRNAs identified by high throughput sequencing in UV-A treatment sRNA library of *Brassica rapa.*

siRNA ID	Sequence (5′-3′)	Length (bp)	Reads Number	siRNA Name [48]	Phase Site in Sequence of BrTAS4 (From 5′ Terminal)
T0111568	uugagcaagauguuggcaugaaau	24	6	TAS D1(+)	675 uugagcaagauguuggcaugaaau 698
T0063765	aauugcagcggugaaggacgagcu	24	11	TAS D2(+)	696 aauugcagcggugaaggacgagcu 719
T0038241	auaacaacgugaaggaucgagguc	24	18	TAS D4(-)	742 gaccucgauccuucacguuguuau 765
T0229627	acgugaaggaucgaggucgaggua	24	3	TAS D4(-)	736 uaccucgaccucgauccuucacgu 759
T0197894	aauaacaacgugaaggaucgaggu	24	4	TAS D4(-)	743 accucgauccuucacguuguuauu 766
T0099445	uuaauuuguaugcaugcugauaac	24	7	TAS D10(+)	885 uuaauuuguaugcaugcugauaac 908
T0159302	uuuguaugcaugcugauaacugaa	24	4	TAS D10(+)	889 uuuguaugcaugcugauaacugaa 912

## Supplementary Materials

The following are available online at https://www.mdpi.com/1422-0067/21/12/4326/s1, Figure S1: The prediction stem-loop structures of premiR828 homologs isolated in Brassica rapa by mfold, Figure S2: Comparison of miR828 sequences in turnip, *Arabidopsis* and tomato, Figure S3: The complementarity binding profiles for BrmiR828 with BrTAS4 and for TAS4-D4 (−) with Bra001917 (BrPAP2), Bra039763 (BrPAP1) and Bra004162 (BrPAP2), Table S1: Primers for premiR828, TAS4 and Real-time PCR primers for detected miR828 and targeted genes, Table S2: Nest primers for RLM 5′-RACE PCR used in this topic. Table S3: Full-length amino acid sequences of MYBs from *Arabidopsis* and *Brassica rapa*.

## Abbreviations

3GT	flavonol-3-glucosyltransferase
ANS	anthocyanidin synthase
Arg	Arginine
Asn	Asparagine
bHLH	basic helix-loop-helix
CYR1	cryptochrome 1
DFR	dihydroflavonol reductase
EGL3	ENHANCER OF GLABRA 3
GL3	GLABRA3
HY5	Elongated Hypocotyl 5
LDOX	leucoanthocyandin dioxygenase
Lys	Lysine
MBW	MYB-bHLH-WD40
miRNAs	microRNAs
MYBL2	MYB-LIKE2
PAP1	Production of Anthocyanin Pigment 1
PIF3	phytochrome-interacting factor 3
qRT-PCR	quantitative reverse transcription PCR
RISC	RNA-induced silencing complex
RLM-5′ RACE	RNA ligasemediated 5′amplification of cDNA ends
Ser	Serine
siRNA	Small interfering RNA
SPL	SQUAMOSA Promoter-Binding Protein-Like
SPL9	SQUAMOSA PROMOTER BINDING PROTEIN-LIKE 9
sRNAs	small RNAs
TAS4	Trans-acting siRNA gene 4
Thr	Threonine
Trp	Tryptophan
TT8	TRANSPARENT TESTA8
TTG1	TRANSPARENT TESTA GLABRA1
UBQ	Ubiquitin
UV-A	ultraviolet A
UVR8	ultraviolet-B receptor UVR8
WDR	WD repeat
