# Supplementary material for: BrmiR828 Targets BrPAP1, BrMYB82, and BrTAS4 Involved in the Light Induced Anthocyanin Biosynthetic Pathway in Brassica rapa
Source: Int J Mol Sci. 2020 Jun 17;21(12):4326. doi: 10.3390/ijms21124326 (PMC7352941; doi:10.3390/ijms21124326)
Supplement: Supplementary file 1 [file ijms-21-04326-s001.pdf]

International Journal of *Molecular Sciences*

MDPI

Supplementary Materials: BrmiR828 targets *BrPAP1*, *BrMYB82* and *BrTAS4* Involved in the Light Induced Anthocyanin Biosynthetic Pathway in *Brassica rapa*

Bo Zhou, Jingtong Leng, Yanyun Ma, Pengzhen Fan, Yuhua Li, Haifang Yan and Qijiang Xu

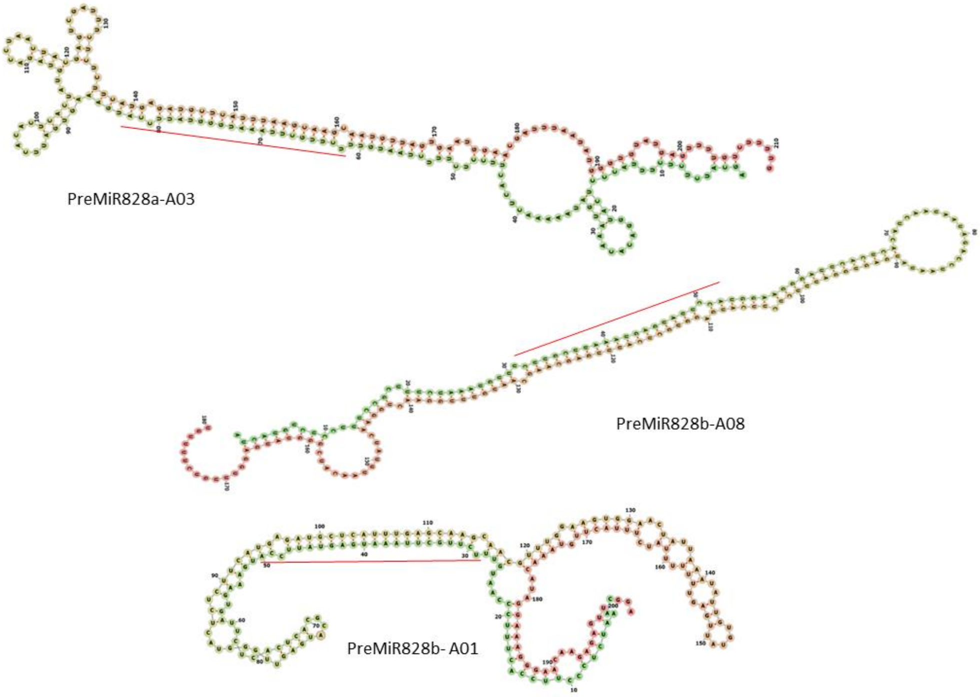

Figure S1. The prediction stem-loop structures of premiR828 homologs isolated in *Brassica rapa* by mfold [51]. The sequence designated by red line is mature miR828 sequence.

At-miR828	UCUUGCUUAAAUGAGUAUUCCA
Br-miR828a	UCUUGCUUAAAUGAGUAUUCCA
Br-miR828b	UCUUGCUUAAAUGGGUAUUCCA
Sl-miR828	UCUUGCUCAAAUGAGUAUUCCA
	******* ***** ********

Figure S2. Comparison of miR828 sequences in turnip, *Arabidopsis* and tomato.

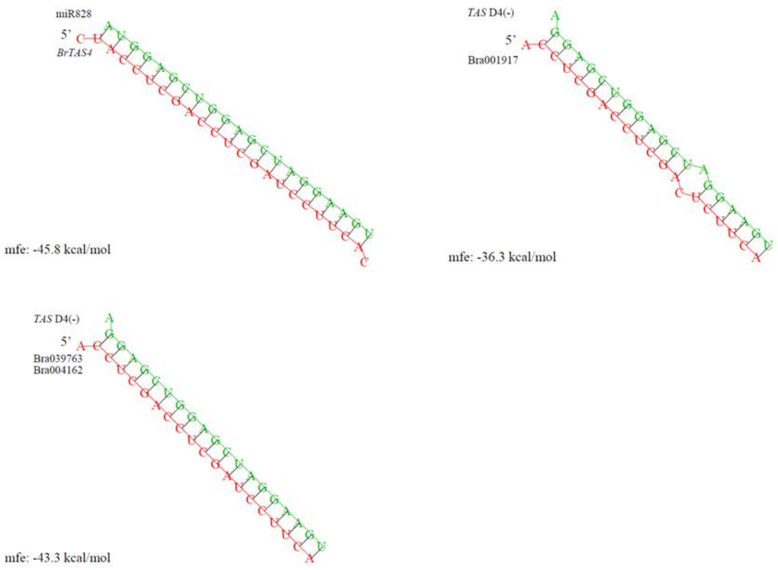

Figure S3. The complementarity binding profiles for BrmiR828 with BrTAS4 and for TAS4-D4 (-) with Bra001917 (BrPAP2), Bra039763 (BrPAP1) and Bra004162 (BrPAP2). Free energies of the duplex structures were calculated using RNA hybrid software (http://bibiserv.techfak.uni-bielefeld.de/rnahybrid, v2.1).

Table S1. Primers for premiR828, TAS4 and Real-time PCR primers for detected miR828 and targeted genes.

Gene ID	Forward primer (5′-3′)	Reverse primer (5′-3′)	Amplification length (nt)
BrTAS4	ATTCCGCGAAAGAGAGCAAGA	ATGCTTCCACCAGATTGTGACT	1150
BramiR828a-A03	AGCATCTCTCTTTACCCTCATGG	CAAAAGACAAAACTCATACACCCA	212
BramiR828b-A01	ACAATCTCCCTTCCACTTTCCC	TCCAACTCTCTTGTTCCCTTTC	203
BramiR828b-A08	ATCATGTCTCCTTTCCGCTTTCT	ACAAAAGACAAACCTCATACCCG	180
MiR828 Stem-loop primer	GTCGTATCCAGTGCAGGGTCCGAGGTATTCGCACTGGATACGACTGGAAT		\
MiR828	CGCCGATCTTGCTTAAATGRGT	CCAGTGCAGGGTCCGAGGTA	65
Bra001917(PAP2)	CCTCCATGCCTTGGACTCAACAACAT	ACTATAAAGTTCATACTTCTCCCCAG	90
Bra004162(PAP2)	CCTCTATGCCTTGGACACAACGATAC	TTCTGGAAAGAGCGCAGCTGGATC	168
Bra039763(PAP1)	GAGCCAAGAACCAGTTGCGATTG	GTTCCACGGTCTCACCATCTAACAGA	113
Bra022602(MYB82)	GGAAGCCACTCCGTTGGTCG	CTATGCGGGCGAAGAAGTTG	128
Bra029113(MYB82)	AGTGGAAGCTCCTACCGACAAGA	GTGGTCGTCATAAGCCAAACAGT	130
BrTAS4-RT	TTCGCAGGTCGATACCAAGT	CCTGAATCTGCATTCACGGT	200
BrUBQ	TTGTAGTCAGCCAAGGTACGACCA	TGGAGAGTTCCGACACCATTGACA	130

Table S2. Nest primers for RLM 5′-RACE PCR used in this topic.

Gene ID	Primer name	Sequence (5′-3′)
Bra001917/Bra004162/Bra039763	Br-PAP1/2-RLM NP1	TCCAAAGTTGCTCAACGTCAAACG
	Br-PAP1/2-RLM NP2	TKGCTCTCMTCTAGCAAACT
	Br-PAP1/2-RLM NP3	AAGRRTCGAGGTCGAGGTTTGA
Bra022602/Bra029113	Br-MYB82-RLM NP1	MMAAGAGTGTCTGKGTAATGAG
	Br-MYB82-RLM NP2	CTATGCGGGCGAAGAAGTTG
	Br-MYB82-RLM NP3	TCCTCCTCCATGGCTTCCTCT
BraTAS4	Br-TAS4-RLM NP1	TGCTTCCACCAGATTGTGACT
	Br-TAS4-RLM NP2	CCTTTTGCCAGTCCCCTTGA

5′ RACE Outer Primer：5′-CATGGCTACATGCTGACAGCCTA-3′. 5′ RACE Inner Primer：5′-CGCGGATCCACAGCCTACTGATGATCAGTCGATG-3′.

Table 3. Full-length amino acid sequences of MYBs from *Arabidopsis* and *Brassica rapa*.

>AtMYB0
MRIRRRDEKENQEYKKGLWTVEEDNILMDYVLNHGTGQWNRIVRKTGLKRCGKSCRLRWMNYLSPNVNK
G
NFTEQEEDLIIRLHKLLGNRWSLIAKRVPGRTDNQVKNYWNTHLSKKLVGDYSSAVKTTGEDDDSPPSLF
ITAATPSSRHHQQENIYENIAKSFNGVVSASYEDKPKQELAQKDVLMATTNDPSHYYGNNALWVHDDDFE
LSSLVMMNFASSDIEYCL
>ATMYB1
MEAEIVRRSEVTGLRREVEESSIGRGDCDGDGGDVGEDAAGFVGTSGRGRRDRVKGPWSKEEDDVLSELV
KRLGARNWSFIARSIPGRSGKSCRLRWCNQLNPNLIRNSFTEVEDQAIIAAHAIHGNKWAVIAKLLPGRT

DNAIKNHWNSALRRRFIDFEKAKNIGTGSLVVDDSGFDRTTTVASSEETLSSGGGCHVTTPIVSPEGKEA
TTSMEMSEEQCVEKTNGEGISRQDDKDPPTLFRPVPRLSSFNACNHMEGSPSPHIQDQNQLQSSKQDAAM
LRLLEGAYSERFVPQTCGGGCCSNNPDGSFQQESLLGPEFVDYLDSPTFPSSELAAIATEIGSLAWLRSG
LESSSVRVMEDAVGRLRPQGSRGHRDHYLVSEQGTNITNVLST
>ATMYB2
MEDYERINSNSPTHEEDSDVRKGPWTEEEDAILVNFVSIHGDARWNHIARSSGLKRTGKSCRLRWLNYLR
PDVRRGNITLEEQFMILKLHSLWGNRWSKIAQYLPGRTDNEIKNYWRTRVQKQAKHLRCDVNSNLFKETM
RNVWMPRLVERINAQSLPTTCEQVESMITDPSQPVNEPSPVEPGFVQFSQNHHQQFVPATELSATSSNSP
AETFSDVRGGVVNGSGYDPSGQTGFGEFNDWGCVGGDNMWTDEESFWFLQDQFCPDTTSYSYN
>ATMYB3
MNKGAWTKEEDQLLVDYIRKHGEGCWRSLPRAAGLQRCGKSCRLRWMNYLRPDLKRGNFTEEEDELIIKL
HSLLGNKWSLIAGRLPGRTDNEIKNYWNTHIKRKLLSRGIDPNSHRLINESVVSPSSLQNDVVETIHLDF
SGPVKPEPVREEIGMVNNCESSGTTSEKDYGNEEDWVLNLELSVGPSYRYESTRKVSVVDSAESTRRWGS
ELFGAHESDAVCLCCRIGLFRNESCRNCRVSDVRTH
>ATMYB4
MGRSPCCEKAHTNKGAWTKEEDERLVAYIKAHGEGCWRSLPKAAGLLRCGKSCRLRWINYLRPDLKRGNF
TEEEDELIIKLHSLLGNKWSLIAGRLPGRTDNEIKNYWNTHIRRKLINRGIDPTSHRPIQESSASQDSKP
IQLEPVTSNTINISFTSAPKVETFHESISFPGKSEKISMLTFKEEKDECPVQEKFPDLNLELRISLPDDV
DRLQGHGKSTTPRCFKCSLGMINGMECRCGRMRCDVVGGSSKGSDMSNGFDFLGLAKKETTSLLGFRSLE
MK
>ATMYB5
MMSCGGKKPVSKKTTPCCTKMGMKRGPWTVEEDEILVSFIKKEGEGRWRSLPKRAGLLRCGKSCRLRWMN
YLRPSVKRGGITSDEEDLILRLHRLLGNRWSLIAGRIPGRTDNEIKNYWNTHLRKKLLRQGIDPQTHKPL
DANNIHKPEEEVSGGQKYPLEPISSSHTDDTTVNGGDGDSKNSINVFGGEHGYEDFGFCYDDKFSSFLNS
LINDVGDPFGNIIPISQPLQMDDCKDGIVGASSSSLGHD
>ATMYB6
MGRSPCCEKAHTNKGAWTKEEDQRLVDYIRNHGEGCWRSLPKSAGLLRCGKSCRLRWINYLRPDLKRGNF
TDDEDQIIIKLHSLLGNKWSLIAGRLPGRTDNEIKNYWNTHIKRKLLSHGIDPQTHRQINESKTVSSQVV
VPIQNDAVEYSFSNLAVKPKTENSSDNGASTSGTTTDEDLRQNGECYYSDNSGHIKLNLDLTLGFGSWSG
RIVGVGSSADSKPWCDPVMEARLSLL
>ATMYB7
MGRSPCCEKEHMNKGAWTKEEDERLVSYIKSHGEGCWRSLPRAAGLLRCGKSCRLRWINYLRPDLKRGNF
THDEDELIIKLHSLLGNKWSLIAARLPGRTDNEIKNYWNTHIKRKLLSKGIDPATHRGINEAKISDLKKT
KDQIVKDVSFVTKFEETDKSGDQKQNKYIRNGLVCKEERVVVEEKIGPDLNLELRISPPWQNQREISTCT
ASRFYMENDMECSSETVKCQTENSSSISYSSIDISSSNVGYDFLGLKTRILDFRSLEMK
>ATMYB8
MGRSPCCEKAHMNKGAWTKEEDQRLIDYIRNHGEGSWRSLPKSVGLLRCGKSCRLRWINYLRPDLKRGNFT
DGEEQIIVKLHSLFGNKWSLIAGKLPGRTDNEIKNYWNTHIKRKLLNRGIDPKTHGSIIEPKTTSFHPRNEDLK
STFPGSVKLKMETSCENCASTSGTTTDEDLRLSVDCDYRYDHLDKELNLDLTLGYSPTRFVGVGSCY
>ATMYB9
MGRSPCCDENGLKKGPWTQEEDDKLIDHIQKHGHGSWRALPKQAGLNRCGKSCRLRWTNY
LRPDIKRGNF
TEEEEQTIINLHSLLGNKWSSIAGNLPGRTDNEIKNYWNTHLRKKLLQMGIDPVTHRPRTDHLNVLAALPQL
IA
AANFNSLLNLNQNVQLDATTLAKAQLLHTMIQVLSTNNNTTNPSFSSSTMQNSNTNLFGQASYLENQNLF
GQSQNFSHILEDENLMVKTQIIDNPLDSFSSPIQPGFQDDHNSLPLLVPASPEESKETQRMIKNKDIVDY
HHHDASNPSSSNSTFTQDHHHPWCDTIDDGASDSFWKEIIEQTCSEPWPFPE
>ATMYB10
MGNRRAPCCDKSQVKRGPWSDEESERLRSFILKNGHQNWRSLPKLAGLMRCGKSCRLRWINYLRPGLKRG
NFTKEEEDTIIHLHQAYGNKWSKIASNFPGRTDNEIKNVWNTHLKKRLVKRSISSSSDVTNHSVSSTSSSSSIS
SVLQDVIIKSERPNQEEEFGEILVEQMACGFEVDAPQSLECLFDDSQVPPPISKPDSLQTHGKSSDHEFWSRLIE
PGFDDYNEWLIFLDNQTC
>ATMYB11
MGRAPCCEKVGIKKGRWTAEEDRTLSDYIQSNGEGSWRSLPKNAGLKRCGKSCRLRWINYLRSDIKRGNITP
EEEDVIVKLHSTLGTRWSTIASNLPGRTDNEIKNYWNSHLSRKLHGYFRKPTVANTVENAPPPPKRRPGRTSR
SAMKPKFILNPKNHKTPNSFKANKSDIVLPTTTIENGEGDKEDALMVLSSSSLSGAEEPGLGPCGYGDDGDC
NPSINGDDGALCLNDDIFDSCFLLDDSHAVHVSSCESNNVKNSEPYGGMSVGHKNIETMADDFVDWDFVW
REGQTLWDEKEDLDSVLSRLLDGEEMESEIRQRDSNDFGEPLDIDEENKMAAWLLS

>ATMYB12
MGRAPCCEKVGIKRGRWTAEEDQILSNYIQSNGEGSWRSLPKNAGLKRCGKSCRLRWINYLRSDLKRGNI
TPEEEELVVKLHSTLGNRWSLIAGHLPGRTDNEIKNYWNSHLSRKLHNFIRKPSISQDVSAVIMTNASSA
PPPPQAKRRLGRTGRSAMKPKIHRTKTRKTKKTSAPPEPNADVAGADKEALMVESSGAEAELGRPCDYYG
DDCNKNLMSINGDNGVLTFDDDIIDLLLDESDPGHLYTNTTCGGDGELHNIRDSEGARGFSDTWNQGNLD
CLLQSCPSVESFLNYDHQVNDASTDEFIDWDCVWQEGSDNNLWHEKENPDSMVSWLLDGDDEATIGNSNC
ENFGEPLDHDDESALVAWLLS
>ATMYB13
MGRRPCCEKIGLKKGPWSAEEDRILINYISLHGHPNWRALPKLAGLLRCGKSCRLRWINY
LRPDIKRGNFTPHEEDTIISLHQLLGNRWSAIAAKLPGRTDNEIKNVWHTHLKKRLHHSQ
DQNNKEDFVSTTAAEMPTSPQQQSSSSADISAITTLGNNNDISNSNKDSATSSEDVLAII
DESFWSEVVLMDCDISGNEKNEKKIENWEGSLDRNDKGYNHDMEFWFDHLTSSSCIIGEM
SDISEF
>ATMYB14
MGRSPCCDKLGLKKGPWTPEEDQKLLAYIEEHGHGSWRSLPEKAGLHRCGKSCRLRWTNYLRPDIKRGKFN
LQEEQTIIQLHALLGNRWSAIATHLPKRTDNEIKNYWNTHLKKRLVKMGIDPVTHKPKNETPLSSLGLSKNA
AILSHTAQWESARLEAEARLARESKLLHLQHYQTKTSSQPHHHHGFTHKSLLPNWTTKPHEDQQQLESPTST
VSFSEMKESIPAKIEFVGSSTGVTLMKEPEHDWINSTMHEFETTQMGEGIEEGFTGLLLGGDSIDRSFSGDKNE
TAGESSGGDCNYYEDNKNYLDSIFNFVDPSPSDSPMF
>ATMYB15
MGRAPCCEKMGLKRGPWTPEEDQILVSFILNHGHSNWRALPKQAGLLRCGKSCRLRWMNYLKPDIKRGNF
TKEEEDAIISLHQILGNRWSAIAAKLPGRTDNEIKNVWHTHLKKRLEDYQPAKPKTSNKKKGTKPKSESVITS
SNSTRSESELADSSNPSGESLFSTSPSTSEVSSMTLISHDGYSNEINMDNKPGDISTIDQECVSFETFGADIDESFW
KETLYSQDEHNYVSNDLEVAGLVEIQQEFQNLGSANNEMIFDSEMDFWFDVLARTGGEQDLLAGL
>ATMYB16
MGRSPCCDKLGLKKGPWTPEEDQKLLAYIEEHGHGSWRSLPEKAGLHRCGKSCRLRWTNYLRPDIKRGKFN
LQEEQTIIQLHALLGNRWSAIATHLPKRTDNEIKNYWNTHLKKRLVKMGIDPVTHKPKNETPLSSLGLSKNA
AILSHTAQWESARLEAEARLARESKLLHLQHYQTKTSSQPHHHHGFTHKSLLPNWTTKPHEDQQQLESPTST
VSFSEMKESIPAKIEFVGSSTGVTLMKEPEHDWINSTMHEFETTQMGEGIEEGFTGLLLGGDSIDRSFSGDKNE
TAGESSGGDCNYYEDNKNYLDSIFNFVDPSPSDSPMF
>ATMYB17
MGRTPCCDKIGLKKGPWTPEEDEVLVAHIKKNGHGSWRTLPKLAGLLRCGKSCRLRWTNYLRPDIKRGPFT
ADEEKLVIQLHAILGNRWAAIAAQLPGRTDNEIKNLWNTHLKKRLLSMGLDPRTHEPLPSYGLAKQAPSSPT
TRHMAQWESARVEAEARLSRESMLFSPSFYSGVVKTECDHFLRIWNSEIGEAFRNLAPLDESTITSQSPCSRAT
STSSALLKSSTNSWGGKEVTVAIHGSDYSPYSNDLEDDSTDSALQLLLDFPISDDDMSFLEENIDSYSQAPPIGL
VSMVSKF
>ATMYB18
MAKTKYGERHRKGLWSPEEDEKLRSFILSYGHSCWTTVPIKAGLQRNGKSCRLRWINYLRPGLKRDMISAEE
EETILTFHSSLGNKWSQIAKFLPGRTDNEIKNYWHSHLKKKWLKSQSLQDAKSISPPSSSSSLVACGKRNPETL
ISNHVFSFQRLLENKSSSPSQESNGNNSHQCSSAPEIPRLFFSEWLSSSYPHTDYSSEFTDSKHSQAPNVEETLSA
YEEMGDVDQFHYNEMMINNSNWTLNDIVFGSKCKKQEHHIYREASDCNSSAEFFSPSTTT
>ATMYB19
MTKSGERPKQRQRKGLWSPEEDQKLKSFILSRGHACWTTVPILAGLQRNGKSCRLRWINYLRPGLKRGSFSEE
EEETILTLHSSLGNKWSRIAKYLPGRTDNEIKNYWHSYLKKRWLKSQPQLKSQISDLTESPSSLLSCGKRNLETE
TLDHVISFQKFSENPTSSPSKESNNNMIMNNSNNLPKLFFSEWISSSNPHIDYSSAFTDSKHINETQDQINEEEV
MMINNNNYSSLEDVMLRTDFLQPDHEYANYYSSGDFFINSDQNYV
>ATMYB20
MGRQPCCDKVGLKKGPWTAEEDRKLINFILTNGQCCWRAVPKLSGLLRCGKSCRLRWTNYLRPDLKRGLLS
DYEEKMVIDLHSQLGNRWSKIASHLPGRTDNEIKNHWNTHIKKKLRKMGIDPLTHKPLSIVEKEDEEPLKKL
QNNTVPFQETMERPLENNIKNISRLEESLGDDQFMEINLEYGVEDVPLIETESLDLICSNSTMSSSTSTSSHSSN
DSSFLKDLQFPEFEWSDYGNSNNDNNNGVDNIIENNMMSLWEISDFSSLDLLLNDESSSTFGLF
>ATMYB21
MEKRGGGSSGGSGSSAEAEVRKGPWTMEEDLILINYIANHGDGVWNSLAKSAGLKRTGKSCRLRWLNYLRP
DVRRGNITPEEQLIIMELHAKWGNRWSKIAKHLPGRTDNEIKNFWRTRIQKYIKQSDVTTTSSVGSHHSSEIN
DQAASTSSHNVFCTQDQAMETYSPTPTSYQHTNMEFNYGNYSAAAVTATVDYPVPMTVDDQTGENYWGM
DDIWSSMHLLNGN
>ATMYB22
MGEPQLFDVPVLEGIKNTTNEIMNQLEDDKMKKTYENKKEASTSKYLKKSDITKKRWTESEDIKLKEMVA

LEPKKWTKVAKHFEGRTPKQCRERWHNHARPNVKKTTWSEEEDQILIEVHKVIGAKWIQISEQLPGRSYN
NVKNHWNTTKRRVQNKSGRTVNRVGNNILENYIRSITINNDDESDGEPTNIENYHDDSEDMLYGEMNLSP
EAITQTTKPLTDASTISPYIPMPKENYTLEVCESLEDYLELLRWWD
>ATMYB23
MRMTRDGKEHEYKKGLWTVEEDKILMDYVRTHGQGHWNRIAKKTGLKRCGKSCRLRWMNYLSPNVNRG
NFTDQEEDLIIRLHKLLGNRWSLIAKRVPGRTDNQVKNYWNTHLSKKLGLGDHSTAVKAACGVESPPSMAL
ITTTSSSHQEISGGKNSTLRFDTLVDESKLKPKSKLVHATPTDVEVAATVPNLFDTFWVLEDDFELSSLTMMDFT
NGYCL
>ATMYB24
MEKRESSGGSGSGDAEVRKGPWTMEEDLILINYIANHGEGVWNSLAKSAGLKRTGKSCRLRWLNYLRPDV
RRGNITPEEQLTIMELHAKWGNRWSKIAKHLPGRTDNEIKNFWRTKIQKYIIKSGETTTVGSQSSEFINH
HATTSHVMNDTQETMDMYSPTTSYQHASNINQQLNYGNYVPESGSIMMPLSVDQSEQNYWSVDDLWPMN
I
YNGN
>ATMYB25
MNGEISRPPELISSRNPCKSFENAIHKAVEAELAELAKSDANGGGKSKVKGPWLPEQDEALTRLVKMCGPRN
WNLISRGIPGRSGKSCRLRWCNQLDPILKRKPFSDEEEHMIMSAQAVLGNKWSVIAKLLPGRTDNAIKNHW
NSNLRRKPAEQWKIPLLMSNTEIVYQLYPSMVRRISNASPKEHLPQEEETGVLSDDKMDDEAKEPPREQNSKT
GVYRPVARMGAFSVCKPGYMAPCEGPLVQASRPDSLAGKFLQSLCYDPIIPSKCGHGCCNHQDSTTLSSSSVL
GSEFVDYEEHSSAELDKELISISNDLNNTAWIRSGKEAEQSLKADDQFRREYAHSKFSGMVNNGVSSQMVRQ
DLRALS
>ATMYB26
MGHHSCCNKQKVKRGLWSPEEDEKLINYINSYGHGCWSSVPKHAGTYTHIHGFCLQRCGKSCRLRWINYLR
PDLKRGSFSPQEAALIIELHSILGNRWAQIAKHLPGRTDNEVKNFWNSSIKKKLMSHHHHGHHHHHLSSMA
SLLTNLPYHNGFNPTTVDDESSRFMSNIITNTNPNFITPSHLSLPSPHVMTPLMFPTSREGDFKFLTTNNPNQS
HHHDNNHYNNLDILSPTPTINNHHQPSLSSCPHDNNLQWPALPDFPASTISGFQETLQDYDDANKLNVFVT
PFNDNAKKLLCGEVLEGKVLSSSSPISQDHGLFLPTTYNFQMTSTSDHQHHHRVDSYINHMIIPSSSSSSPISCG
QYVIT
>ATMYB27
MDFKKEETLRRGPWLEEEDERLVKVISLLGERRWDSLAIVSGLKRSGKSCRLRWMNYLNPTLKRGPMSQEEE
RIIFQLHALWGNKWSKIARRLPGRTDNEIKNYWRTHYRKKQEAQNYGKLFEWRGNTGEELLHKYKETEITR
TKTTSQEHGFVEVVSMESGKEANGGVGGRESFGVMKSPYENRISDWISEISTDQSEANLSEDHSSNSCSENNI
NIGTWWFQETRDFEEFSCSLWS
>ATMYB28
MSRKPCCVGEGLKKGAWTTEEDKKLISYIHDHGEGGWRDIPQKAGLKRCGKSCRLRWTNYLKPEIKRGEFSS
EEEQIIIMLHASRGNKWSVIARHLPRRTDNEIKNYWNTHLKKRLMEQGIDPVTHKPLASSSNPTVDENLNSP
NASSSDKQYSRSSSMPFLSRPPPSSCNMVSKVSELSSNDGTPIQGSSLSCKKRFKKSSSTSRLLNKVAAKATSIKD
ILSASMEGSLSATTISHASFFNGFTEQIRNEEDSSNTSLTNTLAEFDPFSPSSLYPEHEINATSDLNMDQDYDFSQ
FFEKFGGDNHNEENSMNDLLMSDVSQEVSSTSVDDQDNMVGNFEGWSNYLLDHTNFMYDTDSDSLEKHFI
>ATMYB29
MSRKPCCVGEGLKKGAWTAEEDKKLISYIHEHGEGGWRDIPQKAGLKRCGKSCRLRWANYLKPDIKRGEFS
YEEEQIIIMLHASRGNKWSVIARHLPKRTDNEIKNYWNTHLKKLLIDKGIDPVTHKPLAYDSNPDEQSQSGSIS
PKSLPPSSSKNVPEITSSDETPKYDASLSSKKRCFKRSSSTSKLLNKVAARASSMGTILGASIEGTLISSTPLSSCLN
DDFSETSQFQMEEFDPFYQSSEHIIDHMKEDISINNSEYDFSQFLEQFSNNEGEEADNTGGGYNQDLLMSDVS
STSVDEDEMMQNITGWSNYLLDHSDFNYDTSQDYDDKNFI
>ATMYB30
MVRPPCCDKGGVKKGPWTPEEDIILVTYIQEHGPGNWRAVPTNTGLLRCSKSCRLRWTNYLRPGIKRGNF
TEHEEKMIVHLQALLGNRWAAIASYLPQRTDNDIKNYWNTHLKKKLNKVNQDSHQELDRSSLSSSPSSSS
ANSNSNISRGKWERRLQTDIHLAKKALSEALSPAVAPIITSTVTTTSSSAESRRSTASASGFLRTQETST
TYASSTENIAKLLKGWVKNSPKTQNSADQIASTEVKEVIKSDDGKECAGAFQSFSEFDHSYQQAGVSPDH
ETKPDITGCCSNQSQWSLFEKWLFEDSGGQIGDILLDENTNFF
>ATMYB31
MGRPPCCEKIEVKKGPWTPEEDIILVSYIQQHGPGNWRSVPANTGLLRCSKSCRLRWTNYLRPGIKRGNFTQP
EEKMIIHLQALLGNRWAAIASYLPQRTDNDIKNYWNTHLKKKLVMMKFQNGIINENKTNLATDISSCNNNN
NGCNHNKRTTNKGQWEKKLQTDINMAKQALFQALSLDQPSSLIPPDPDSPKPHHHSTTTYASSTDNISKLLQ
NWTSSSSSKPNTSSVSNNRSSSPGEGGLFDHHSLFSSNSESGSVDEKLNLMSETSMFKGESKPDIDMEATPTTTT
TDDQGSLSLIEKWLFDDQGLVQCDDSQEDLIDVSLEELK
>ATMYB32

MGRSPCCEKDHTNKGAWTKEEDDKLISYIKAHGEGCWRSLPRSAGLQRCGKSCRLRWINYLRPDLKRGNFT
LEEDDLIIKLHSLLGNKWSLIATRLPGRTDNEIKNYWNTHVKRKLLRKGIDPATHRPINETKTSQDSSDSSKTE
DPLVKILSFGPQLEKIANFGDERIQKRVEYSVVEERCLDLNLELRISPPWQDKLHDERNLRFGRVKYRCSACRF
GFGNGKECSCNNVKCQTEDSSSSSYSSTDISSSIGYDFLGLNNTRVLDFSTLEMK
>ATMYB33
MSYTSTDSDHNESPAADDNGSDCRSRWDGHALKKGPWSSAEDDILIDYVNKHGEGNWNAVQKHTSLFRC
GKSCRLRWANHLRPNLKKGAFSQEEEQLIVELHAKMGNRWARMAAHLPGRTDNEIKNYWNTRIKRRQRA
GLPLYPPEMHVEALEWSQEYAKSRVMGEDRRHQDFLQLGSCESNVFFDTLNFTDMVPGTFDLADMTAYKN
MGNCASSPRYENFMTPTIPSSKRLWESELLYPGCSSTIKQEFSSPEQFRNTSPQTISKTCSFSVPCDVEHPLYGNR
HSPVMIPDSHTPTDGIVPYSKPLYGAVKLELPSFQYSETTFDQWKKSSSPPHSDLLDPFDTYIQSPPPPTGGEESD
LYSNFDTGLLDMLLLEAKIRNNSTKNNLYRSCASTIPSADLGQVTVSQTKSEEFDNSLKSFLVHSEMSTQNAD
ETPPRQREKKRKPLLDITRPDVLLASSWLDHGLGIVKETGSMSDALAVLLGDDIGNDYMNMSVGASSGVGSC
SWSNMPPVCQMTELP
>ATMYB34
MVRTPCCKEEGIKKGAWTPEEDQKLIAYLHLHGEGGWRTLPEKAGLKRCGKSCRLRWANYLRPDIKRGEFSP
EEDDTIIKLHALKGNKWAAIATSLAGRTDNEIKNYWNTNLKKRLKQKGIDAITHKPINSTGQTGFEPKVNKP
VYSSGSARLLNRVASKYAVELNRDLLTGIISGNSTVAEDSQNSGDVDSPTSTLLNKMAATSVLINTTTTYSGFSD
NCSFTDEFNEFFNNEEISDIYTTVDNFGFMEELKSILSYGDASAGVIENSPEVNVADAMEFIDSWNEDDNMVG
VFV
>ATMYB35
MGRPPCCDKSNVKKGLWTEEEDAKILAYVAIHGVGNWSLIPKKAGLNRCGKSCRLRWTNYLRPDLKHDSFS
TQEEELIIECHRAIGSRWSSIARKLPGRTDNDVKNHWNTKLKKKLMKMGIDPVTHKPVSQLLAEFRNISGHG
NASFKTEPSNNSILTQSNSAWEMMRNTTTNHESYYTNSPMMFTNSSEYQTTPFHFYSHPNHLLNGTTSSCSSS
SSSTSITQPNQVPQTPVTNFYWSDFLLSDPVPQVVGSSATSDLTFTQNEHHFNIEAEYISQNIDSKASGTCHSAS
SFVDEILDKDQEMLSQFPQLLNDFDY
>ATMYB36
MGRAPCCDKANVKKGPWSPEEDVKLKDYIDKYGTGGNWIALPQKIGLKRCGKSCRLRWLNYLRPNIKHGG
FSEEEDRIILSLYISIGSRWSIIAAQLPGRTDNDIKNYWNTKLKKKLLGRQKQMNRQDSITDSTENNLSNNNNN
KSPQNLSNSALERLQLHMQLQNLQSPFSSFYNNPILWPKLHPLLQSTTTNQNPKLASQESFHPLGVNVDHQ
HNNTKLAQINNGASSLYSENVEQSQNPAHEFQPNFGFSQDLRLDNHNMDFMNRGVSKELFQVGNEFELTN
GSSWWSEEVELERKTTSSSSWGSASVLDQTTEGMVMLQDYAQMSYHSV
>ATMYB37
MGRAPCCDKTKVKRGPWSPEEDSKLRDYIEKYGNGGNWISFPLKAGLRRCGKSCRLRWLNYLRPNIKHGDF
SEEEDRIIFSLFAAIGSRWSIIAAHLPGRTDNDIKNYWNTKLRKKLLSSSSDSSSSAMASPYLNPISQDVKRPTSP
TTIPSSSYNPYAENPNQYPTKSLISSINGFEAGDKQIISYINPNYPQDLYLSDSNNNTSNANGFLLNHNMCDQY
KNHTSFSSDVNGIRSEIMMKQEEIMMMMMIDHHIDQRTKGYNGEFTQGYYNYYNGHGDLKQMISGTGTNS
NINMGGSGSSSSSISNLAENKSSGSLLLEYKCLPYFYS
>ATMYB38
MGRAPCCDKANVKRGPWSPEEDAKLKDYIEKQGTGGNWIALPHKAGLRRCGKSCRLRWLNYLRPNIRHG
DFTEEEDNIIYSLFASIGSRWSVIAAHLQGRTDNDIKNYWNTKLKKKLIATMAPPPHHHLAIATSSSSASPSSSS
HYNMINSLLPYNPSTNQLLTPHQGIMMTMMGQQQQLFYQEDMGNLVNSPNRNNLIMSHQEDNQEQSTNK
GIMLLSDVRSGSSTTSTVTRVKMEHRDHDDHHHHHEEDERSMTSVVMEDYGMEEIKQLISSSCTSSNNSLWF
DENKTEDKFMLYY
>ATMYB39
MEEEEKNPSSIYIVADLLEDIFLRLPLKSILISKSVSKRWRSILESKTFVERRMSLQKKRKILAAYNCKCGWEPRLL
PGSSQCKGNEEIVYLHCNAAQPSFTCDGLVCILEPRWIDVLNPWTRQLRRYGFGFGTIFGVGSAFSPRHWAM
GFGKDKVTGSYKVVKMCLISFSEICARDPEVEYSVLDVETGEWRMLSPPPYKVFEVRKSECANGSIYWLHKPT
ERAWTILALDLHKEELHNISVPDMSVTQETFQIVNLEDRLAIANTYTKTEWKLEIWSMDTEVETWTKTYSIDL
ENRVASRERRNRWFTPVSVSKQGNIVFYDNHKRLFKYYPRKNEILYLSADTCVISPFFENLAPLPQKSTLHTPII
AGEPNAPLTTLVVVGSIWSPLFLFSVKL
>ATMYB40
MCDLLACLRKISMGRKPCCDKIGLKRGPWTIEEDHRLMNFILNNGIHCWRIVPKLAGLLRCGKSCRLRWINY
LRPDLKRGGFTDAEEDRIMELHSQLGNRWSKIASHFSGRTDNEIKNHWNTKIKKKMKHLGLDPATHKPMN
DITHQTDPNQDKKPNMCSTINEGEEIKDQTPKDDVITETTKTLMLSDNDEELVAKNCKILCAEEVDLESLFET
QCNEISSSSFSSLCSNISRSESSSYLAEDSISLEQWDLDMTDPFVPWDLFANLDDNLFLL
>ATMYB41
MGRSPCCDKNGVKKGPWTAEEDQKLIDYIRFHGPGNWRTLPKNAGLHRCGKSCRLRWTNYLRPDIKRGRF
SFEEEETIIQLHSVMGNKWSAIAARLPGRTDNEIKNHWNTHIRKRLVRSGIDPVTHSPRLDLLDLSSLLSALFN

QPNFSAVATHASSLLNPDVLRLASLLLPLQNPNPVYPSNLDQNLQTPNTSSESSQPQAETSTVPTNYETSSLEP
MNARLDDVGLADVLPPLSESFDLDSLMSTPMSSPRQNSIEAETNSSTFFDFGIPEDFILDDFMF
>ATMYB42
MGRQPCCDKLMVKKGPWTAEEDKKLINFILTNGHCCWRALPKLAGLRRCGKSCRLRWTNYLRPDLKRGLL
SDAEEQLVIDLHALLGNRWSKIAARLPGRTDNEIKNHWNTHIKKKLLKMEIDPSTHQPLNKVFTDTNLVD
KSETSSKADNVNDNKIVEIDGTTTNTIDDSIITHQNSSNDDYELLGDIIHNYGDLFNILWTNDEPPLVDD
ASWSNHNVGIGGTAAVAASDKNNTAAEEDFPERSFEKQNGESWMFLDYCQEFGVEDFGFECYHGFGQSSM
KTGHKD
>ATMYB43
MGRQPCCDKVGLKKGPWTIEEDKKLINFILTNGHCCWRALPKLSGLLRCGKSCRLRWINYLRPDLKRGLL
SEYEEQKVINLHAQLGNRWSKIASHLPGRTDNEIKNHWNTHIKKKLRKMGIDPLTHKPLSEQEASQQAQG
RKKSLVPHDDKNPKQDQQTKDEQEQHQLEQALEKNNTSVSGDGFCIDEVPLLNPHEILIDISSSHHHHSN
DDNVNINTSKFTSPSSSSSSTSSCISSVVPGDEFSKFFDEMEILDLKWLSSDDSLGDDISKDGKFNNSTV
DTMNLWDINDLSSLDMFMNEHDDGFIGNGNGCSRMVLDQDSWTFDLL
>ATMYB44
MADRIKGPWSPEEDEQLRRLVVKYGPRNWTVISKSIPGRSGKSCRLRWCNQLSPQVEHRPFSAEEDETIARAH
AQFGNKWATIARLLNGRTDNAVKNHWNSTLKRKCGGYDHRGYDGSEDHRPVKRSVSAGSPPVVTGLYMSP
GSPTGSDVSDSSTIPILPSVELFKPVPRPGAVVLPLPIETSSSSDDPPTSLSLSLPGADVSEESNRSHESTNINNTTSS
RHNHNNTVSFMPFSGGFRGAIEEMGKSFPGNGGEFMAVVQEMIKAEVRSYMTEMQRNNGGGFVGGFIDN
GMIPMSQIGVGRIE
>ATMYB45
MVFKSEKSNREMKSKEKQRKGLWSPEEDEKLRSHVLKYGHGCWSTIPLQAGLQRNGKSCRLRWVNYLRPGL
KKSLFTKQEETILLSLHSMLGNKWSQISKFLPGRTDNEIKNYWHSNLKKGVTLKQHETTKKHQTPLITNSLEA
LQSSTERSSSSINVGETSNAQTSSFSPNLVFSEWLDHSLLMDQSPQKSSYVQNLVLPEERGFIGPCGPRYLGNDS
LPDFVPNSEFLLDDEISSEIEFCTSFSDNFLFDGLINELRPM
>ATMYB46
MRKPEVAIAASTHQVKKMKKGLWSPEEDSKLMQYMLSNGQGCWSDVAKNAGLQRCGKSCRLRWINYLRP
DLKRGAFSPQEEDLIIRFHSILGNRWSQIAARLPGRTDNEIKNFWNSTIKKRLKKMSDTSNLINNSSSSPNTASD
SSSNSASSLDIKDIIGSFMSLQEQGFVNPSLTHIQTNNPFPTGNMISHPCNDDFTPYVDGIYGVNAGVQGELYF
PPLECEEGDWYNANINNHLDELNTNGSGNAPEGMRPVEEFWDLDQLMNTEVPSFYFNFKQSI
>ATMYB47
MGRTTWFDVDGMKKGEWTAEEDQKLGAYINEHGVCDWRSLPKRAGLQRCGKSCRLRWLNYLKPGIRRGK
FTPQEEEEIIQLHAVLGNRWAAMAKKMQNRTDNDIKNHWNSCLKKRLSRKGIDPMTHEPIIKHLTVNTTNA
DCGNSSTTTSPSTTESSPSSGSSRLLNKLAAGISSRQHSLDRIKYILSNSIIESSDQAKEEEEKEEEEEERDSMMGQ
KIDGSEGEDIQIWGEEEVRRLMEIDAMDMYEMTSYDAVMYESSHILDHLF
>ATMYB48
MKMMQEEGNRKGPWTEQEDILLVNFVHLFGDRRWDFIAKVSGLNRTGKSCRLRWVNYLHPGLKRGKMTP
QEERLVLELHAKWGNRWSKIARKLPGRTDNEIKNYWRTHMRKKAQEKKRPVSPTSSFSNCSSSSVTTTTTNT
QDTSCHSRKSSGEVSFYDTGGSRSTREMNQENEDVYSLDDIWREIDHSAVNIIKPVKDIYSEQSHCLSYPNLAS
PSWESSLDSIWNMDADKSKISSYFANDQFPFCFQHSRSPWSSG
>ATMYB49
MGKSSSSEESEVKKGPWTPEEDEKLVGYIQTHGPGKWRTLPKNAGLKRCGKSCRLRWTNYLRPDIKRGEFSL
QEEETIIQLHRLLGNKWSAIAIHLPGRTDNEIKNYWNTHIKKKLLRMGIDPVTHCPRINLLQLSSFLTSSLFKS
MSQPMNTPFDLTTSNINPDILNHLTASLNNVQTESYQPNQQLQNDLNTDQTTFTGLLNSTPPVQWQNNGEY
LGDYHSYTGTGDPSNNKVPQAGNYSSAAFVSDHINDGENFKAGWNFSSSMLAGTSSSSSTPLNSSSTFYVNG
GSEDDRESFGSDMLMFHHHHDHNNNALNLS
>ATMYB50
MKRHSCCYKQKLRKGLWSPEEDEKLLNYITKHGHGCWSSVPKLAGLERCGKSCRLRWINYLRPDLKRGAFS
SEEQNLIVELHAVLGNRWSQIAARLPGRTDNEIKNLWNSCIKKKLMKKGIDPITHKPLSEVGKETNRSDNNN
STSFSSETNQDLFVKKTSDFAEYSAFQKEESNSVSLRNSLSSMIPTQFNIDDGSVSNAGFDTQVCVKPSIILLPPP
NNTSSTVSGQDHVNVSEPNWESNSGTTSHLNNPGMEEMKWSEEYLNESLFSTQVYVKSETDFNSNIAFPWS
QSQACDVFPKDLQRMAFSFGGQTL
>ATMYB51
MVRTPCCKAELGLKKGAWTPEEDQKLLSYLNRHGEGGWRTLPEKAGLKRCGKSCRLRWANYLRPDIKRGE
FTEDEERSIISLHALHGNKWSAIARGLPGRTDNEIKNYWNTHIKKRLIKKGIDPVTHKGITSGTDKSENL
PEKQNVNLTTSDHDLDNDKAKKNNKNFGLSSASFLNKVANRFGKRINQSVLSEIIGSGGPLASTSHTTNT
TTTSVSVDSESVKSTSSSFAPTSNLLCHGTVATTPVSSNFDVDGNVNLTCSSSTFSDSSVNNPLMYCDNF
VGNNNVDDEDTIGFSTFLNDEDFMMLEESCVENTAFMKELTRFLHEDENDVVDVTPVYERQDLFDEIDNY

FG
>ATMYB52
MMCSRGHWRPAEDEKLRELVEQFGPHNWNAIAQKLSGRSGKSCRLRWFNQLDPRINRNPFTEEEEERLLA
PHRIHGNRWSVIARFFPGRTDNAVKNHWHVIMARRGRERSKLRPRGLGHDGTVAATGMIGNYKDCDKERR
LATTTAINFPYQFSHINHFQVLKESLTGKIGFRNSTTPIQEGAIDQTKRPMEFYNFLQVNTDSKIHELID
NSRKDEEEDVDQNNRIRNENCVPFFDFLSVGNSASQGLC
>ATMYB53
MGRSPSSDETGLKKGPWLPEEDDKLINYIHKHGHSSWSALPKLAGLNRCGKSCRLRWTNYLRPDIKRGKFSA
EEEETILNLHAVLGNKWSMIASHLPGRTDNEIKNFWNTHLKKKLIQMGFDPMTHQPRTDDIFSSLSQLMSLS
NLRGLVDLQQQFPMEDQALLNLQTEMAKLQLFQYLLQPSPAPMSINNINPNILNLLIKENSVTSNIDLGFLSS
HLQDFNNNNLPSLKTLDDNHFSQNTSPIWLHEPPSLNQTMLPTHDPCAQSVDGFGSNQASSSHDQEVAVTD
SVDWPDHHLFDDSMFPDISYQS
>ATMYB54
MIMCSRGHWRPAEDEKLKDLVEQYGPHNWNAIALKLPGRSGKSCRLRWFNQLDPRINRNPFTEEEEERLL
AAHRIHGNRWSIIARLFPGRTDNAVKNHWHVIMARRTRQTSKPRLLPSTTSSSSLMASEQIMMSSGGYNH
NYSSDDRKKIFPADFINFPYKFSHINHLHFLKEFFPGKIALSHKANQSKKPMEFYNFLQVNTDSNKSEII
DQDSGQSKRSDSDTKHESHVPFFDFLSVGNSAS
>ATMYB55
MGRHSCCYKQKLRKGLWSPEEDEKLLRYITKYGHGCWSSVPKQAGTFLFIQIHLLFGLQRCGKSCRLRWINY
LRPDLKRGAFSQDEENLIIELHAVLGNRWSQIAAQLPGRTDNEIKNLWNSCLKKKLRLRGIDPVTHKLLTEIET
GTDDKTKPVEKSQQTYLVETDGSSSTTTCSTNQNNNTDHLYTGNFGFQRLSLENGSRIAAGSDLGIWIPQTGR
NHHHHVDETIPSAVVLPGSMFSSGLTGYRSSNLGLIELENSFSTGPMMTEHQQIQESNYNNSTFFGNGNLNW
GLTMEENQNPFTISNHSNSSLYSDIKSETNFFGTEATNVGMWPCNQLQPQQHAYGHI
>ATMYB56
MNPNLLEKDLRGKETTNGSIRYKEANNFRSLPNSHTAACKTSLNNPSISRNHPHNKSASVLESEDEHGNERG
ENEKSLRMRGKSGINTKVCSRGHWRPTEDAKLKELVAQFGPQNWNLISNHLLGRSGKSCRLRWFNQLDPRI
NKRAFTEEEEFRLLAAHRAYGNKWALISRLFPGRTDNAVKNHWHVIMARRTRESQRQRQQPPPTLSRDAEM
TVSSSCRYNQGKFINEEDDDDDVSAVSTCTTELSLTPPSSAYQPRFFNYDSTLASGKDGQCVQRAEVNGIYGK
KMDHQNHHTISVSERKVEMKMRSGYYYFDFLGVGAS
>ATMYB57
METTMKKKGRVKATITSQKEEEGTVRKGPWTMEEDFILFNYILNHGEGLWNSVAKASGLKRTGKSCRLRWL
NYLRPDVRRGNITEEEQLLIIQLHAKLGNRWSKIAKHLPGRTDNEIKNFWRTKIQRHMKVSSENMMNHQH
HCSGNSQSSGMTTQGSSGKAIDTAESFSQAKTTTFNVVEQQSNENYWNVEDLWPVHLLNGDHHVI
>ATMYB58
MGKGRAPCCDKTKVKRGPWSHDEDLKLISFIHKNGHENWRSLPKQAGLLRCGKSCRLRWINYLRPDVKRG
NFSAEEEDTIIKLHQSFGNKWSKIASKLPGRTDNEIKNVWHTHLKKRLSSETNLNADEAGSKGSLNEEENSQE
SSPNASMSFAGSNISSKDDDAQISQMFEHILTYSEFTGMLQEVDKPELLEMPFDLDPDIWSFIDGSDSFQQPEN
RALQESEEDEVDKWFKHLESELGLEENDNQQQQQQHKQGTEDEHSSSLLESYELLIH
>ATMYB59
MKLVQEEYRKGPWTEQEDILLVNFVHLFGDRRWDFVAKVSGLNRTGKSCRLRWVNYLHPGLKRGKMTPQE
ERLVLELHAKWGNRWSKIARKLPGRTDNEIKNYWRTHMRKKAQEKKRPMSPTSSSSNCCSSSMTTTTTQD
TGGSNGKMNQEGEDGYYSMDDIWREIDQSGHVIKPVKDNYYSEQSCYLNFPPLASPTWESSLESIWNMDA
DESKMSSFAIDQFPLSFEHGSGRL
>ATMYB60
MGRPPCCDKIGIKKGPWTPEEDIILVSYIQEHGPGNWRSVPTNTGLLRCSKSCRLRWTNYLRPGIKRGNF
TPHEEGMIIHLQALLGNKWASIASYLPQRTDNDIKNYWNTHLKKKLNKSDSDERSRSENIALQTSSTRNT
INHRSTYASSTENISRLVEGWMRASPKSSTSTTFLEHKMQNRTNNFIDHHSDQFPYEQLQGSREEGHSKG
INGDDDQGIKNSENNNGDDVHHEDGDHEDDDDHNATPPLTFIEKWLLEETSTTGGQMEEMSHLMELSNM
L
>ATMYB61
MGRHSCCYKQKLRKGLWSPEEDEKLLTHITNHGHGCWSSVPKLAGLQRCGKSCRLRWINYLRPDLKRGAFS
PEEENLIVELHAVLGNRWSQIASRLPGRTDNEIKNLWNSSIKKKLKQRGIDPNTHKPISEVESFSDKDKPTTSN
NKRSGNDHKSPSSSSATNQDFFLERPSDLSDYFGFQKLNFNSNLGLSVTTDSSLCSMIPPQFSPGNMVGSVLQT
PVCVKPSISLPPDNNSSSPISGGDHVKLAAPNWEFQTNNNNTSNFFDNGGFSWSIPNSSTSSSQVKPNHNFEEI
KWSEYLNTPFFIGSTVQSQTSQPIYIKSETDYLANVSNMTDPWSQNENLGTTETSDVFSKDLQRMAVSFGQSL
>ATMYB62
MENSMKKKKSFKESEDEELRRGPWTLEEDTLLTNYILHNGEGRWNHVAKCAGLKRTGKSCRLRWLNYLKP
DIRRGNLTPQEQLLILELHSKWGNRWSKIAQYLPGRTDNEIKNYWRTRVQKQARQLNIESNSDKFFDAVRSF

WVPRLIEKMEQNSSTTTTYCCPQNNNNNSLLLPSQSHDSLSMQKDIDYSGFSNIDGSSSTSTCMSHLTTVPHF
MDQSNTNIIDGSMCFHEGNVQEFGGYVPGMEDYMVNSDISMECHVADGYSAYEDVTQDPMWNVDDIWQF
RE
>ATMYB63
MGKGRAPCCDKTKVKRGPWSPEEDIKLISFIQKFGHENWRSLPKQSGLLRCGKSCRLRWINYLRPDLKRGNF
TSEEEETIIKLHHNYGNKWSKIASQLPGRTDNEIKNVWHTHLKKRLAQSSGTADEPASPCSSDSVSRGKDDKS
SHVEDSLNRETNHRNELSTSMSSGGSNQQDDPKIDELRFEYIEEAYSEFNDIIIQEVDKPDLLEIPFDSDPDIWSF
LDTSNSFQQSTANENSSGSRATTEEESDEDEVKKWFKHLESELGLEEDDNQQQYKEEESSSSLLKNYELMIH
>ATMYB64
MEEQKIQEKSLAHGAAPPLTAVERFLNGQKNEALCFKKQERSIDRPIVKTTRAIEIRNENKENMMFGPRKEK
NLAVIGEIVVKGAAKDYTCKDITKKQPYKNIIKGQWTADEDRKLIKLVMQHGERKWAVISEKLEGRAGKQCR
ERWHNHLRPDIKKDSWSEEEERLLVEAHTRIGNKWAEIAKLIQGRTENSIKNHWNATKRRQNSKRKHKRSK
NADSNSDIDDLSPSAKRPRILEDYIKNIENNDKNNGENIMTTSGNNVLSTSNYDQFNSEDSTSSLLDDPYDEE
LVFLKNIFENHSLENINLSQGTEITQSSSSGFMIENPKPKPNLYNNTFGTHLGAMVTEPANSSHLASDIYLSDLL
NGTASSSSSLTFLSSNNNEHAGENELLLPQANSTSERREMDLIEMLSGSTQGSNIWFPLF
>ATMYB65
MSYTTATADSDDGMHSSIHNESPAPDSISNGCRSRGKRSVLKKGPWTSTEDGILIDYVKKHGEGNWNAVQK
HTSLARCGKSCRLRWANHLRPNLKKGAFSQEEEQLIVEMHAKMGNKWAQMAEHLPGRTDNEIKNYWNT
RIKRRQRAGLPLYPPEIYVDDLHWSEEYTKSNIIRVDRRRRHQDFLQLGNSKDNVLFDDLNFAASLLPAASDL
SDLVACNMLGTGASSSRYESYMPPILPSPKQIWESGSRFPMCSSNIKHEFQSPEHFQNTAVQKNPRSCSISPCDV
DHHPYENQHSSHMMMVPDSHTVTYGMHPTSKPLFGAVKLELPSFQYSETSAFDQWKTTPSPPHSDLLDSVD
AYIQSPPPSQVEESDCFSSCDTGLLDMLLHEAKIKTSAKHSLLMSSPQKSFSSTTCTTNVTQNVPRGSENLIKSG
EYEDSQKYLGRSEITSPSQLSAGGFSSAFAGNVVKTEELDQVWEPKRVDITRPDVLLASSWLDQGCYGIVSDTS
SMSDALALLGGDDIGNSYVTVGSSSGQAPRGVGSYGWTNMPPVWSL
>ATMYB66
MRKKVSSSGDEGNNEYKKGLWTVEEDKILMDYVKAHGKGHWNRIAKKTGLKRCGKSCRLRWMNYLSPNV
KRGNFTEQEEDLIIRLHKLLGNRWSLIAKRVPGRTDNQVKNYWNTHLSKKLGIKDQKTKQSNGDIVYQINLP
NPTETSEETKISNIVDNNNILGDEIQEDHQGSNYLSSLWVHEDEFELSTLTNMMDFIDGHCF
>ATMYB67
MREKWEMKRDEMGHRCCGKHKVKRGLWSPEEDEKLLRYITTHGHPSWSSVPKLAGLQRCGKSCRLRWINY
LRPDLRRGSFNEEEEQIIIDVHRILGNKWAQIAKHLPGRTDNEVKNFWNSCIKKKLLSQGLDPSTHNLMPSH
KRSSSSNNNNIPKPNKTTSIMKNPTDLDQSTTAFSITNINPPTSTKPNKLKSPNQTTIPSQTVIPINDNMSSTQT
MIPINDPMSSLLDDENMIPHWSDVDGMAIHEAPMLPSDKAVVGVDDDDLNMDILFNTPSSSAFDPDFASIFS
SAMSIDFNPMDDLGSWTF
>ATMYB68
MGRAPCCDKANVKKGPWSPEEDAKLKDYIENSGTGGNWIALPQKIGLRRCGKSCRLRWLNYLRPNIKHGG
FSEEEDNIICNLYVTIGSRWSIIAAQLPGRTDNDIKNYWNTRLKKKLLNKQRKEFQEARMKQEMVMMKRQ
QQGQGQGQSNGSTDLYLNNMFGSSPWPLLPQLPPPHHQIPLGMMEPTSCNYYQTTPSCNLEQKPLITLKN
MVKIEEEQERTNPDHHHQDSVTNPFDFSFSQLLLDPNYYLGSGGGGEGDFAIMSSSTNSPLPNTSSDQHP
SQQQEILQWFGSSNFQTEAINDMFINNNNNNNIVNLETIENTKVYGDASVAGAAVRAALAGGTTSTSADQ
STISWEDITSLVNSEDASYFNAPNHV
>ATMYB69
MEMSRGSNSFDNKKPSCQRGHWRPVEDDNLRQLVEQYGPKNWNFIAQHLYGRSGKSCRLRWYNQLDPNIT
KKPFTEEEEERLLKAHRIQGNRWASIARLFPGRTDNAVKNHFHVIMARRKRENFSSTATSTFNQTWHTVLSPS
SSLTRLNRSHFGLWRYRKDKSCGLWPYSFVSPPTNGQFGSSSVSNVHHEIYLERRKSKELVDPQNYTFHAATP
DHKMTSNEDGPSMGDDGEKNDVTFIDFLGVGLAS
>ATMYB70
MSGSTRKEMDRIKGPWSPEEDDLLQSLVQKHGPRNWSLISKSIPGRSGKSCRLRWCNQLSPEVEHRGFTAEED
DTIILAHARFGNKWATIARLLNGRTDNAIKNHWNSTLKRKCSGGGGGGEEGQSCDFGGNGGYDGNLTDEK
PLKRRASGGGGVVVVTALSPTGSDVSEQSQSSGSVLPVSSSCHVFKPTARAGGVVIESSSPEEEEKDPMTCLRLS
LPWVNESTTPPELFPVKREEEEEKEREISGLGGDFMTVVQEMIKTEVRSYMADLQLGNGGGAGGGASSCMV
QGTNGRNVGFREFIGLGRIE
>ATMYB71
MSLWGGMGGGWGMVEEGWRKGPWTAEEDRLLIDYVQLHGEGRWNSVARLAGLKRNGKSCRLRWVNYL
RPDLKRGQITPHEETIILELHAKWGNRWSTIARSLPGRTDNEIKNYWRTHFKKKTKSPTNSAEKTKNRILKRQ
QFQQQRQMELQQEQQLLQFNQIDMKKIMSLLDDDNNNGDNTFSSSSSGESGALYVPHQITHSTTTSGCEPNS
NGYYPVVPVTIPEANVNEDNAIWDGLWNLDFEGQGSFGGAACAPRKHYFQNMVIPFC
>ATMYB72

MGKGRAPCCDKNKVKRGPWSPQEDLTLITFIQKHGHQNWRSLPKLAGLLRCGKSCRLRWINYLRPDVKRG
NFSKKEEDAIIHYHQTLGNKWSKIASFLPGRTDNEIKNVWNTHLKKRLTPSSSSSLSSTHDQSTKADHDKNC
DGAQEEIHSGLNESQNSATSSHHQGECMHTKPELHEVNGLNEIQFLLDHDDFDDITSEFLQDNDILFPLDSLL
HNHQTHISTQEMTREVTKSQSFDHPQPDIPCGFEDTNEESDLRRQLVESTTPNNEYDEWFNFIDNQTYFDDF
NFVGEVCL
>ATMYB73
MSNPTRKNMERIKGPWSPEEDDLLQRLVQKHGPRNWSLISKSIPGRSGKSCRLRWCNQLSPEVEHRAFSQEE
DETIIRAHARFGNKWATISRLLNGRTDNAIKNHWNSTLKRKCSVEGQSCDFGGNGGYDGNLGEEQPLKRTA
SGGGGVSTGLYMSPGSPSGSDVSEQSSGGAHVFKPTVRSEVTASSSGEDPPTYLSLSLPWTDETVRVNEPVQLN
QNTVMDGGYTAELFPVRKEEQVEVEEEEAKGISGGFGGEFMTVVQEMIRTEVRSYMADLQRGNVGGSSSGG
GGGGSCMPQSVNSRRVGFREFIVNQIGIGKME
>ATMYB74
MGRSPCCEKKNGLKKGPWTPEEDQKLIDYINIHGYGNWRTLPKNAGLQRCGKSCRLRWTNYLRPDIKRGRF
SFEEEETIIQLHSIMGNKWSAIAARLPGRTDNEIKNYWNTHIRKRLLKMGIDPVTHTPRLDLLDISSILSSSIYNS
SHHHHHHHQQHMNMSRLMMSDGNHQPLVNPEILKLATSLFSNQNHPNNTHENNTVNQTEVNQYQTGY
NMPGNEELQSWFPIMDQFTNFQDLMPMKTTVQNSLSYDDDCSKSNFVLEPYYSDFASVLTTPSSSPTPLNSSS
STYINSSTCSTEDEKESYYSDNITNYSFDVNGFLQFQ
>ATMYB75
MEGSSKGLRKGAWTTEEDSLLRQCINKYGEGKWHQVPVRAGLNRCRKSCRLRWLNYLKPSIKRGKLSSDEV
DLLLRLHRLLGNRWSLIAGRLPGRTANDVKNYWNTHLSKKHEPCCKIKMKKRDITPIPTTPALKNNVYKPRP
RSFTVNNDCNHLNAPPKVDVNPPCLGLNINNVCDNSIIYNKDKKKDQLVNNLIDGDNMWLEKFLEESQEV
DILVPEATTTEKGDTLAFDVDQLWSLFDGETVKFD
>ATMYB76
MSKRPYCIGEGLKKGAWTTEEDKKLISYIHDHGEGGWRDIPEKAGLKRCGKSCRLRWTNYLKPDIKRGEFSY
EEEQIIIMLHASRGNKWSVIARHLPKRTDNEVKNYWNTHLKKRLIDDGIDPVTHKPLASSNPNPVEPMKFDF
QKKSNQDEHSSQSSSTTPASLPLSSNLNSVKSKISSGETQIESGHVSCKKRFGRSSSTSRLLNKVAARASSIGNILS
TSIEGTLRSPASSSGLPDSFSQSYEYMIDNKEDLGTSIDLNIPEYDFPQFLEQLINDDDENENIVGPEQDLLMSDF
PSTFVDEDDILGDITSWSTYLLDHPNFMYESDQDSDEKNFL
>ATMYB77
MADRVKGPWSQEEDEQLRRMVEKYGPRNWSAISKSIPGRSGKSCRLRWCNQLSPEVEHRPFSPEEDETIVTA
RAQFGNKWATIARLLNGRTDNAVKNHWNSTLKRKCSGGVAVTTVTETEEDQDRPKKRRSVSFDSAFAPVDT
GLYMSPESPNGIDVSDSSTIPSPSSPVAQLFKPMPISGGFTVVPQPLPVEMSSSSEDPPTSLSLSLPGAENTSSSHN
NNNNALMFPRFESQMKINVEERGEGRRGEFMTVVQEMIKAEVRSYMAEMQKTSGGFVVGGLYESGGNGGF
RDCGVITPKVE
>ATMYB78
MGDKGRSLKINKNMEEFTKVEEEMDVRRGPWTVEEDLELINYIASHGEGRWNSLARCAELKRTGKSCRLRW
LNYLRPDVRRGNITLEEQLLILELHTRWGNSNEFDHKFSSSCEDRWSKIAQYLPGRTDNEIKNYWRTRVQKH
AKQLKCDVNSQQFKDTMKYLWMPRLVERIQAASIGSVSMSSCVTTSSDQFVINNNNTNNVDNLALMSNPN
GYITPDNSSVAVSPVSDLTECQVSSEVWKIGQDENLVDPKMTSPNYMDNSSGLLNGDFTKMQDQSDLNWFE
NINGMVPNYSDSFWNIGNDEDFWLLQQHQQVHDNGSF
>ATMYB79
MVEEVWRKGPWTAEEDRLLIEYVRVHGEGRWNSVSKLAGLKRNGKSCRLRWVNYLRPDLKRGQITPHEESII
LELHAKWGNRWSTIARSLPGRTDNEIKNYWRTHFKKKAKPTTNNAEKIKSRLLKRQHFKEQREIELQQEQQ
LFQFDQLGMKKIISLLEENNSSSSDGGGDVFYYPDQITHSSKPFGYNSNSLEEQLQGRFSPVNIPDANTMNED
NAIWDGFWNMDVVNGHGGNLGVVAATAACGPRKPYFHNLVIPFC
>ATMYB80
MGRIPCCEKENVKRGQWTPEEDNKLASYIAQHGTRNWRLIPKNAGLQRCGKSCRLRWTNYLRPDLKHGQF
SEAEEHIIVKFHSVLGNRWSLIAAQLPGRTDNDVKNYWNTKLKKKLSGMGIDPVTHKPFSHLMAEITTTLNP
PQVSHLAEAALGCFKDEMLHLLTKKRVDLNQINFSNHNPNPNNFHEIADNEAGKIKMDGLDHGNGIMKL
WDMGNGFSYGSSSSSFGNEERNDGSASPAVAAWRGHGGIRTAVAETAAAEEEERRKLKGEVVDQEEIGSEGG
RGDGMTMMRNHHHHQHVFNVDNVLWDLQADDLINHMV
>ATMYB81
MGKVRQDSGSDDDNSIKKSFTKGPWTQAEDNLLIAYVDKHGDGNWNAVQNNSGLSRCGKSCRLRWVNHL
RPDLKKGAFTEKEEKRVIELHALLGNKWARMAEELPGRTDNEIKNFWNTRLKRLQRLGLPVYPDEVREHAM
NAATHSGLNTDSLDGHHSQEYMEADTVEIPEVDFEHLPLNRSSSYYQSMLRHVPPTNVFVRQKPCFFQPPNV
YNLIPPSPYMSTGKRPREPETAFPCPGGYTMNEQSPRLWNYPFVENVSEQLPDSHLLGNAAYSSPPGPLVHGV
ENFEFPSFQYHEEPGGWGADQPNPMPEHESDNTLVQSPLTAQTPSDCPSSSLYDGLLESVVYGSSGEKPATDT
DSESSLFQSFTPANENITGKTCFLTLYALHALHCLCNQFKKSPLLHLHDKLNWCNKFRFNSFKSGTHIL

>ATMYB82
MECKREEGKSYVKRGLWKPEEDMILKSYVETHGEGNWADISRRSGLKRGGKSCRLRWKNYLRPNIKRGSMS
PQEQDLIIRMHKLLGNRWSLIAGRLPGRTDNEVKNYWNTHLNKKPNSRRQNAPESIVGATPFTDKPVMSTE
LRRSHGEGGEEESNTWMEETNHFGYDVHVGSPLPLISHYPDNTLVFDPCFSFTDFFPLL
>ATMYB83
MMMRKPDITTIRDKGKPNHACGGNNNKPKLRKGLWSPDEDEKLIRYMLTNGQGCWSDIARNAGLLRCGK
SCRLRWINYLRPDLKRGSFSPQEEDLIFHLHSILGNRWSQIATRLPGRTDNEIKNFWNSTLKKRLKNNSNNNT
SSGSSPNNSNSNSLDPRDQHVDMGGNSTSLMDDYHHDENMMTVGNTMRMDSSSPFNVGPMVNSVGLNQ
LYDPLMISVPDNGYHQMGNTVNVFSVNGLGDYGNTILDPISKRVSVEGDDWFIPPSENTNVIACSTSNNLNL
QALDPCFNSKNLCHSESFKVGNVLGIENGSWEIENPKIGDWDLDGLIDNNSSFPFLDFQVD
>ATMYB84
MGRAPCCDKANVKKGPWSPEEDAKLKSYIENSGTGGNWIALPQKIGLKRCGKSCRLRWLNYLRPNIKHGGF
SEEEENIICSLYLTIGSRWSIIAAQLPGRTDNDIKNYWNTRLKKKLINKQRKELQEACMEQQEMMVMMKRQH
QQQQIQTSFMMRQDQTMFTWPLHHHNVQVPALFMNQTNSFCDQEDVKPVLIKNMVKIEDQELEKTNPHH
HQDSMTNAFDHLSFSQLLLDPNHNHLGSGEGFSMNSILSANTNSPLLNTSNDNQWFGNFQAETVNLFSGAS
TSTSADQSTISWEDISSLVYSDSKQFF
>ATMYB85
MGRQPCCDKLGVKKGPWTVEEDKKLINFILTNGHCCWRALPKLAGLRRCGKSCRLRWTNYLRPDLKRGLL
SHDEEQLVIDLHANLGNKWSKIASRLPGRTDNEIKNHWNTHIKKKLLKMGIDPMTHQPLNQEPSNIDNSKTI
PSNPDDVSVEPKTTNTKYVEISVTTTEEESSSTVTDQNSSMDNENHLIDNIYDDDELFSYLWSDETTKDEASWS
DSNFGVGGTLYDHNISGADADFPIWSPERINDEKMFLDYCQDFGVHDFGF
>ATMYB86
MGRHSCCFKQKLRKGLWSPEEDEKLLNYITRHGHGCWSSVPKLAGLQRCGKSCRLRWINYLRPDLKRGAFS
QDEESLIIELHAALGNRWSQIATRLPGRTDNEIKNFWNSCLKKKLRRKGIDPTTHKPLITNELQSLNVIDQKLT
SSEVVKSTGSINNLHDQSMVVSSQQGPWWFPANTTTTNQNSAFCFSSSNTTTVSDQIVSLISSMSTSSSPTPMTS
NFSPAPNNWEQLNYCNTVPSQSNSIYSAFFGNQYTEASQTMNNNNPLVDQHHHHQDMKSWASEILHYTE
HNQSSETVIEAEVKPDIANYYWRSASSSSPNQEAATLLHDANVEVYGKNLQKLNNMVFDQSL
>ATMYB87
MGRAPCCDKMAVKKGPWSTEEDAVLKSYIEKHGTGNNWISLPQRIGIKRCGKSCRLRWLNYLRPNLKHGGF
TDEEDYIICSLYITIGSRWSIIASQLPGRTDNDIKNYWNTRLKKKLLSKQGKAFHQQLNVKFERGTTSSSSSQNQ
IQIFHDENTKSNQTLYNQVVDPSMRAFAMEEQSMIKNQILEPFSWEPNKVLFDVDYDAAASSYHHHASPSLN
SMSSTSSIGTNNSSLQMSHYTVNHNDHDQPDMFFMDGFENFQAELFDEIANNNTVENGFDGTEILINNNYL
DHDISSFIDYPLYDNE
>ATMYB88
MEETTKQNNMKKKKKILLHSDDSKKKERHIVTWSPEEDDILRKQISLQGTENWAIIASKFNDKSTRQCRR
RWYTYLNSDFKRGGWSPEEDTLLCEAQRLFGNRWTEIAKVVSGRTDNAVKNRFTTLCKKRAKHEAMAKEN
RIACCVNSDNKRLLFPDGISTPLKVESESPLTKKMR
>ATMYB89
MYLFMYKCNIVLEETHVFQNTPCDVSLQRPFNGFGENNALPLRKMHQEEKKKKHRGGHWTLSEDLKLKEL
VAVFGPQNWKFIGEKMEPRTSLSCRQRWFNQLDPKINKRNFTDEEEEKLLRAHILYGNKWSKIAKLFNRR
TDHAVKNHWHSLMNRIIRKQSASDIRSFDNIQNYQTSNFLPGLCLLNTKQ
>ATMYB90
MEGSSKGLRKGAWTAEEDSLLRLCIDKYGEGKWHQVPLRAGLNRCRKSCRLRWLNYLKPSIKRGRLSNDEV
DLLLRLHKLLGNRWSLIAGRLPGRTANDVKNYWNTHLSKKHESSCCKSKMKKKNIISPPTTPVQKIGVFKPR
PRSFSVNNGCSHLNGLPEVDLIPSCLGLKKNNVCENSITCNKDDEKDDFVNNLMNGDNMWLENLLGENQE
ADAIVPEATTAEHGATLAFDVEQLWSLFDGETVELD
>ATMYB91
MKERQRWSGEEDALLRAYVRQFGPREWHLVSERMNKPLNRDAKSCLERWKNYLKPGIKKGSLTEEEQRLVI
RLQEKHGNKWKKIAAEVPGRTAKRLGKWWEVFKEKQQREEKESNKRVEPIDESKYDRILESFAEKLVKERSN
VVPAAAAAATVVMANSNGGFLHSEQQVQPPNPVIPPWLATSNNGNNVVARPPSVTLTLSPSTVAAAAPQPP
IPWLQQQQPERAENGPGGLVLGSMMPSCSGSSESVFLSELVECCRELEEGHRAWADHKKEAAWRLRRLELQL
ESEKTCRQREKMEEIEAKMKALREEQKNAMEKIEGEYREQLVGLRRDAEAKDQKLADQWTSRHIRLTKFLE
QQMGCRLDRP
>ATMYB92
MGRSPISDDSGLKKGPWTPDEDEKLVNYVQKHGHSSWRALPKLAGLNRCGKSCRLRWTNYLRPDIKRGRFS
PDEEQTILNLHSVLGNKWSTIANQLPGRTDNEIKNFWNTHLKKKLIQMGFDPMTHRPRTDIFSGLSQLMSLS
SNLRGFVDLQQQFPIDQEHTILKLQTEMAKLQLFQYLLQPSSMSNNVNPNDFDTLSLLNSIASFKETSNNTTS
NNLDLGFLGSYLQDFHSLPSLKTLNSNMEPSSVFPQNLDDNHFKFSTQRENLPVSPIWLSDPSSTTPAHVNDD

LIFNQYGIEDVNSNITSSSGQESGASASAAWPDHLLDDSIFSDIP
>ATMYB93
MGRSPCCDENGLKKGPWTPEEDQKLIDYIHKHGHGSWRALPKLADLNRCGKSCRLRWTNYLRPDIKRGKF
SAEEEQTILHLHSILGNKWSAIATHLQGRTDNEIKNFWNTHLKKKLIQMGIDPVTHQPRTDLFASLPQLIALA
NLKDLIEQTSQFSSMQGEAAQLANLQYLQRMFNSSASLTNNNGNNFSPSSILDIDQHHAMNLLNSMVSWN
KDQNPAFDPVLELEANDQNQDLFPLGFIIDQPTQPLQQQKYHLNNSPSELPSQGDPLLDHVPFSLQTPLNSE
DHFIDNLVKHPTDHEHEHDDNPSSWVLPSLIDNNPKTVTSSLPHNNPADASSSSSYGGCEAASFYWPDICFD
ESLMNVIS
>ATMYB94
MGRPPCCDKIGVKKGPWTPEEDIILVSYIQEHGPGNWRSVPTHTGLRRCSKSCRLRWTNYLRPGIKRGNFTEH
EEKMILHLQALLGNRWAAIASYLPERTDNDIKNYWNTHLKKKLKKMNDSCDSTINNGLDNKDFSISNKNTT
SHQSSNSSKGQWERRLQTDINMAKQALCDALSIDKPQNPTNFSIPDLGYGPSSSSSSTTTTTTTRNTNPYPSG
VYASSAENIARLLQNFMKDTPKTSVPLPVAATEMAITTAASSPSTTEGDGEGIDHSLFSFNSIDEAEEKPKLIDH
DINGLITQGSLSLFEKWLFDEQSHDMIINNMSLEGQEVLF
>ATMYB95
MGRTTWFDVDGLRKGEWTAEEDRKLVVYINEHGLGEWGSLPKRAGLQRCGKSCRLRWLNYLRPGIKRGKF
TPQEEEEIIKYHALLGNRWAAIAKQMPNRTDNDIKNHWNSCLKKRLAKKGIDPMTHEPTTTTSLTVDVTSSS
TTSSPTPSPTSSSFSSCSSTGSARFLNKLAAGISSRKHGLESIKTVILAEQPREAVDEEKMMTINMKEKELISCYME
IDETMSIDELPCDDSTSGFVAFDDYSLIDPYRDGVYVSDFYDETEHLDLFLL
>ATMYB96
MGRPPCCEKIGVKKGPWTPEEDIILVSYIQEHGPGNWRSVPTHTGLRRCSKSCRLRWTNYLRPGIKRGNFTEH
EEKTIVHLQALLGNRWAAIASYLPERTDNDIKNYWNTHLKKKLKKINESGEEDNDGVSSSNTSSQKNHQST
NKGQWERRLQTDINMAKQALCEALSLDKPSSTLSSSSSLPTPVITQQNIRNFSSALLDRCYDPSSSSSSTTTTTS
NTTNPYPSGVYASSAENIARLLQDFMKDTPKALTLSSSSPVSETGPLTAAVSEEGGEGFEQSFFSFNSMDETQN
LTQETSFFHDQVIKPEITMDQDHGLISQGSLSLFEKWLFDEQSHEMVGMALAGQEGMF
>ATMYB97
MIVYGGGASEDGEGGGVVLKKGPWTVAEDETLAAYVREYGEGNWNSVQKKTWLARCGKSCRLRWANHL
RPNLRKGSFTPEEERLIIQLHSQLGNKWARMAAQLPGRTDNEIKNYWNTRLKRFQRQGLPLYPPEYSQNNH
QQQMYPQQPSSPLPSQTPASSFTFPLLQPPSLCPKRCYNTAFSPKASYISSPTNFLVSSPTFLHTHSSLSSYQSTNP
VYSMKHELSSNQIPYSASLGVYQVSKFSDNGDCNQNLNTGLHTNTCQLLEDLMEEAEALADSFRAPKRRQI
MAALEDNNNNNNFFSGGFGHRVSSNSLCSLQGLTPKEDESLQMNTMQDEDITKLLDWGSESEEISNGQSSVI
TTENNLVLDDHQFAFLFPVDDDTNNLPGIC
>ATMYB98
MENFVDENGFASLNQNIFTRDQEHMKEEDFPFEVVDQSKPTSFLQDFHHLDHDHQFDHHHHHGSSSSHPL
LSVQTTSSCINNAPFEHCSYQENMVDFYETKPNLMNHHHFQAVENSYFTRNHHHHQEINLVDEHDDPMDL
EQNNMMMMRMIPFDYPPTETFKPMNFVMPDEISCVSADNDCYRATSFNKTKPFLTRKLSSSSSSSSWKETKKS
TLVKGQWTAEEDRVLIQLVEKYGLRKWSHIAQVLPGRIGKQCRERWHNHLRPDIKKETWSEEEDRVLIEFHK
EIGNKWAEIAKRLPGRTENSIKNHWNATKRRQFSKRKCRSKYPRPSLLQDYIKSLNMGALMASSVPARGRRR
ESNNKKKDVVVAVEEKKKEEEVYGQDRIVPECVFTDDFGFNEKLLEEGCSIDSLLDDIPQPDIDAFVHGL
>ATMYB99
MGGRKPCCDEVGLRKGPWTVEEDGKLVDFLRARGNCGGGGGGWCWRDVPKLAGLRRCGKSCRLRWTNY
LRPDLKRGLFTEEEIQLVIDLHARLGNRWSKIAVELPGRTDNDIKNYWNTHIKRKLIRMGIDPNTHRRFDQQK
VNEEETILVNDPKPLSETEVSVALKNDTSAVLSGNLNQLADVDGDDQPWSFLMENDEGGGGDAAGELTMLL
SGDITSSCSSSSSLWMKYGEFGYEDLELGCFDV
>ATMYB100
MKKNYQKKNIKVVSTSKYLKKSDIDKVNWTESEDIKLKEIMALGPKNKWTKVAKKFEGRTGKQCRERWYN
HARPNIKKTAWSEEEDQILIEAHKVLGTKWVEIAQQLPGRSDNNIKNHWNTTKRRVQNKRGGTVNPVGNN
ILENYIRCITINNEDFLKTDGSYGEPTNIESDDDSKDMLYGEMNLSLETITTQTTKPLTNASTTSPYVQMPEDNY
TMEDCESLEDILELLRWWE
>ATMYB101
MDGGGETTATATMEGRGLKKGPWTTTEDAILTEYVRKHGEGNWNAVQKNSGLLRCGKSCRLRWANHLRP
NLKKGSFTPDEEKIIIDLHAKLGNKWARMASQLPGRTDNEIKNYWNTRMKRRQRAGLPLYPHEIQHQGIDID
DEFEFDLTSFQFQNQDLDHNHQNMIQYTNSSNTSSSSSFSSSSSQPSKRLRPDPLVSTNPGLNPIPDSSMDFQM
FSLYNNSLENDNNQFGFSVPLSSSSSSNEVCNPNHILEYISENSDTRNTNKKDIDAMSYSSLLMGDLEIRSSSFP
LGLDNSVLELPSNQRPTHSFSSSPIIDNGVHLEPPSGNSGLLDALLEESQALSRGGLFKDVRVSSSDLCEVQDK
RVKMDFENLLIDHLNSSNHSSLGANPNIHNKYNEPTMVKVTVDDDDELLTSLLNNFPSTTTPLPDWYRVTE
MQNEASYLAPPSGILMGNHQGNGRVEPPTVPPSSSVDPMASLGSCYWSNMPSIC
>ATMYB102

MARSPCCEKNGLKKGPWTSEEDQKLVDYIQKHGYGNWRTLPKNAGLQRCGKSCRLRWTNYLRPDIKRGRF
SFEEEETIIQLHSFLGNKWSAIAARLPGRTDNEIKNFWNTHIRKKLLRMGIDPVTHSPRLDLLDISSILASSLYNS
SSHHMNMSRLMMDTNRRHHQQHPLVNPEILKLATSLFSQNQNQNLVVDHDSRTQEKQTVYSQTGVNQYQ
TNQYFENTITQELQSSMPPFPNEARQFNNMDHHFNGFGEQNLVSTSTTSVQDCYNPSFNDYSSSNFVLDPSYS
DQSFNFANSVLNTPSSSPSPTTLNSSYINSSSCSTEDEIESYCSNLMKFDIPDFLDVNGFII
>ATMYB103
MGHHSCCNQQKVKRGLWSPEEDEKLIRYITTHGYGCWSEVPEKAGLQRCGKSCRLRWINYLRPDIRRGRFSP
EEEKLIISLHGVVGNRWAHIASHLPGRTDNEIKNYWNSWIKKKIRKPHHHYSRHQPSVTTVTLNADTTSIATT
IEASTTTTSTIDNLHFDGFTDSPNQLNFTNDQETNIKIQETFFSHKPPLFMVDTTLPILEGMFSENIITNNNKNN
DHDDTQRGGRENVCEQAFLTTNTEEWDMNLRQQEPFQVPTLASHVFNNSSNSNIDTVISYNLPALIEGNVD
NIVHNENSNVQDGEMASTFECLKRQELSYDQWDDSQQCSNFFFWDNLNINVEGSSLVGNQDPSMNLGSSA
LSSSFPSSF
>ATMYB104
MIQDQANDLLAMKKTFTKSKWKPEEDRILKDYVIQYGDRTWTHVPKRTGLPHNPASCRFRWMNHLKPSLK
KGPFTDEEEKRVLQLHAVLGNKWSQMAREFPGRTDNEIKNFWNARRMRLKGKGLPVYPDEVREQAIRTAA
QYGVKVELLNAHYSQDSLMAGNVEKPQELNNLALNQCSPYYQSTLANVQPSRNRVMEPETTFPFTGGSAM
NEQNPTLLCNPYVESTQEQLPDSHLFGNVTYSSPPMPLIHEVENLELPSFQGFDFHEEPSSFGAEQYNPMLNL
EPHNTLVQSPLIGQTPTDFPSSFYDELLDELLESVVNGSLGEIPKTDTSSESQLFQSSLRSHTDATPDIANTTGYV
GSNERNTTNDDDWIRLLLDEGFI
>ATMYB105
MEMVHADVASLSITPCFPSSLSSSSHHHYNQQQHCIMSEDQHHSMDQTTSSDYFSLNIDNAQHLRSYYTSHR
EEDMNPNLSDYSNCNKKDTTVYRSCGHSSKASVSRGHWRPAEDTKLKELVAVYGPQNWNLIAEKLQGRSG
KSCRLRWFNQLDPRINRRAFTEEEEERLMQAHRLYGNKWAMIARLFPGRTDNSVKNHWHVIMARKFREQS
SSYRRRKTMVSLKPLINPNPHIFNDFDPTRLALTHLASSDHKQLMLPVPCFPGYDHENESPLMVDMFETQM
MVGDYIAWTQEATTFDFLNQTGKSEIFERINEEKKPPFFDFLGLGTV
>ATMYB106
MPIHVRDREKGRLQNLNRDIFCCVSPSIYQSDAKRAAFVIILIMIISPCCDKAGLKKGPWTPEEDQKLLAYIEE
HGHGSWRSLPEKAGLQRCGKSCRLRWTNYLRPDIKRGKFTVQEEQTIIQLHALLGNRWSAIATHLPKRTDNE
IKNYWNTHLKKRLIKMGIDPVTHKHKNETLSSSTGQSKNAATLSHMAQWESARLEAEARLARESKLLHLQH
YQNNNNLNKSAAPQQHCFTQKTSTNWTKPNQGNGDQQLESPTSTVTFSENLLMPLGIPTDSSRNRNNNNN
ESSAMIELAVSSSTSSDVSLVKEHEHDWIRQINCGSGGIGEGFTSLLIGDSVGRGLPTGKNEATAGVGNESEYN
YYEDNKNYWNSILNLVDSSPSDSATMF
>ATMYB107
MGRSPCCDESGLKKGPWTPEEDQKLINHIRKHGHGSWRALPKQAGLNRCGKSCRLRWTNYLRPDIKRGNF
TAEEEQTIINLHSLLGNKWSSIAGHLPGRTDNEIKNYWNTHIRKKLIQMGIDPVTHRPRTDHLNVLAALPQL
LAAANFNNLLNLNQNIQLDATSVAKAQLLHSMIQVLSNNNTSSSFDIHHTTNNLFGQSSFLENLPNIENPYD
QTQGLSHIDDQPLDSFSSPIRVVAYQHDQNFIPPLISTSPDESKETQMMVKNKEIMKYNDHTSNPSSTSTFTQD
HQPWCDIIDDEASDSYWKEIIEQTCSEPWPFRE
>ATMYB108
MDEKGRSLKNNNMEDEMDLKRGPWTAEEDFKLMNYIATNGEGRWNSLSRCAGLQRTGKSCRLRWLNYLR
PDVRRGNITLEEQLLILELHSRWGNRWSKIAQYLPGRTDNEIKNYWRTRVQKHAKQLKCDVNSQQFKDTM
KYLWMPRLVERIQSASASSAAAATTTTTTTTGSAGTSSCITTSNNQFMNYDYNNNNMGQQFGVMSNNDYIT
PENSSVAVSPASDLTEYYSAPNPNPEYYSGQMGNSYYPDQNLVSSQLLPDNYFDYSGLLDEDLTAMQEQSNLS
WFENINGAASSSDSLWNIGETDEEFWFLQQQQQFNNNGSF
>ATMYB109
MEGETHQSEPLPLASGDSDEGISAAIEAELAELAAGDSSGGGGCGGGGGGIRSKVKGPWSTEEDAVLTKLVR
KLGPRNWSLIARGIPGRSGKSCRLRWCNQLDPCLKRKPFSDEEDRMIISAHAVHGNKWAVIAKLLTGRTDNA
IKNHWNSTLRRKYADLWNNGQWMANSVTTASVKNENVDETTNPPSSKQQLPQGDINSSPPKPPQVSDVVM
EEAANEPQEPQEQQEQAPPVVSNVPTENNVFRPVARVGAFSIYNPTSQKNGYRDYNIVPCEGPLIQAAKPDS
LAGKFLQSLCDEPQIPSKCGHGCSTLPAETKFSRNSVLGPEFVDYEEPSAVFNQELISIATDLNNIAWIKSGLDN
AVVREAEQSLKMDNYNYNDPRIKFTGMMPRQDFFCARS
>ATMYB110
MKMDFSCFQEYPFEFHCRGTTFNGFRENNAVSETVEEFCNKRRMQKKSDDLKTKKKKKQSVSRVCSRGHW
RISEDTQLMELVSVYGPQNWNHIAESMQGRTGKSCRLRWFNQLDPRINKRAFSDEEEERLLAAHRAFGNKW
AMIAKLFNGRTDNALKNHWHVLMARKMRQQSSSYVQRFNGSAHESNTDHKIFNLSPGNVDDDEDVNLK
KCSWEMLKEGTTNLKAQYLQEEYSSSRMPMQGPHHHYSTFPADSLALTLHVSIQEPSSSSSLSLPSSSTTGEHT
MVTRYFETIKPPAFIDFLGVGH
>ATMYB111

MGRAPCCEKIGLKRGRWTAEEDEILTKYIQTNGEGSWRSLPKKAGLLRCGKSCRLRWINYLRRDLKRGNITS
DEEEIIVKLHSLLGNRWSLIATHLPGRTDNEIKNYWNSHLSRKIYAFTAVSGDGHNLLVNDVVLKKSCSSSSGA
KNNNKTKKKKKGRTSRSSMKKHKQMVTASQCFSQPKELESDFSEGGQNGNFEGESLGPYEWLDGELERLLS
SCVWECTSEEAVIGVNDEKVCESGDNSSCCVNLFEEEQGSETKIGHVGITEVDHDMTVEREREGSFLSSNSNE
NNDKDWWVGLCNSSEVGFGVDEELLDWEFQGNVTCQSDDLWDLSDIGEITLE
>ATMYB112
MNISRTEFANCKTLINHKEEVEEVEKKMEIEIRRGPWTVEEDMKLVSYISLHGEGRWNSLSRSAGLNRTGKSC
RLRWLNYLRPDIRRGDISLQEQFIILELHSRWGNRWSKIAQHLPGRTDNEIKNYWRTRVQKHAKLLKCDVNS
KQFKDTIKHLWMPRLIERIAATQSVQFTSNHYSPENSSVATATSSTSSSEAVRSSFYGGDQVEFGTLDHMTNGG
YWFNGGDTFETLCSFDELNKWLIQ
>ATMYB113
MGESPKGLRKGTWTTEEDILLRQCIDKYGEGKWHRVPLRTGLNRCRKSCRLRWLNYLKPSIKRGKLCSDEVD
LVLRLHKLLGNRWSLIAGRLPGRTANDVKNYWNTHLSKKHDERCCKTKMINKNITSHPTSSAQKIDVLKPR
PRSFSDKNSCNDVNILPKVDVVPLHLGLNNNYVCESSITCNKDEQKDKLININLLDGDNMWWESLLEADVL
GPEATETAKGVTLPLDFEQIWARFDEETLELN
>ATMYB114
MEGSSKGLRKGAWTAEEDSLLRQCIGKYGEGKWHQVPLRAGLNRCRKSCRLRWLNYLKPSIKRGKFSSDEV
DLLLRLHKLLGNRWSLIAGRLPGRTANDVKNYWNTHLSKKHEPCCKTKIKRINIITPPNTPAQKVDIF
>ATMYB115
MYHQNLISSTPNQNSNPHDWDIQNPLFSIHPSAEIPSKYPFMGITSCPNTNVFEEFQYKITNDQNFPTTYNTPF
PVISEGISYNMHDVQENTMCGYTAHNQGLIIGCHEPVLVHAVVESQQFNVPQSEDINLVSQSERVTEDKVMF
KTDHKKKDIIGKGQWTPTEDELLVRMVKSKGTKNWTSIAKMFQGRVGKQCRERWHNHLRPNIKKNDWSE
EEDQILIEVHKIVGNKWTEIAKRLPGRSENIVKNHWNATKRRLHSVRTKRSDAFSPRNNALENYIRSITINNN
ALMNREVDSITANSEIDSTRCENIVDEVMNLNLHATTSVYVPEQAVLTWGYDFTKCYEPMDDTWMLMNGW
N
>ATMYB116
MSNITKKKCNGNEEGAEQRKGPWTLEEDTLLTNYISHNGEGRWNLLAKSSGKSCRLRWLNYLKPDIKRGN
LTPQEQLLILELHSKWGNRWSKISKYLPGRTDNDIKNYWRTRVQKQARQLNIDSNSHKFIEVVRSFWFPR
LINEIKDNSYTNNIKANAPDLLGPILRDSKDLGFNNMDCSTSMSEDLKKTSQFMDFSDLETTMSLEGSRG
GSSQCVSEVYSSFPCLEEEYMVAVMGSSDISALHDCHVADSKYEDDVTQDLMWNMDDIWQFNEYAHFN
>ATMYB117
MFITEKQVWMDEIVARRASSSWDFPFNDINIHQHHHRHCNTSHEFEILKSPLGDVAVHEEESNNNNPNFSNS
ESGKKETTDSGQSWSSSSSKPSVLGRGHWRPAEDVKLKELVSIYGPQNWNLIAEKLQGRSGKSCRLRWFNQL
DPRINRRAFTEEEEERLMQAHRLYGNKWAMIARLFPGRTDNSVKNHWHVVMARKYREHSSAYRRRKLMSN
NPLKPHLTNNHHPNPNPNYHSFISTNHYFAQPFPEFNLTHHLVNNAPITSDHNQLVLPFHCFQGYENNEPP
MVVSMFGNQMMVGDNVGATSDALCNIPHIDPSNQEKPEPNDAMHWIGMDAVDEEVFEKAKQQPHFFDF
LGLGTA
>ATMYB118
MEFESVFKMHYPYLAAVIYDDSSTLKDFHPSLTDDFSCVHNVHHKPSMPHTYEIPSKETIRGITPSPCTE
AFEACFHGTSNDHVFFGMAYTTPPTIEPNVSHVSHDNTMWENDQNQGFXFGTESTLNQAMXDSNQFNMP
K
PLLSANEDTIMNRRQNNQVMIKTEQIKKKNKRFQMRRICKPTKKASIIKGQWTPEEDKLLVQLVDLHGTK
KWSQIAKMLQGRVGKQCRERWHNHLRPDIKKDGWTEEEDIILIKAHKEIGNRWAEIARKLPGRTENTIKN
HWNATKRRQHSRRTKGKDEISLSLGSNTLQNYIRSVTYNDDPFMTANANANIGPRNMRGKGKNVMVAVSE
YDEGECKYIVDGVNNLGLEDGRIKMPSLAAMSASGSASTSGSASGSGSGVTMEIDEPMTDSWMVMHGCDE
VMMNEIALLEMIAHGRL
>ATMYB119
MEDRRLVHGAAPPLTAVERFLYGQKNDALCSKKQESSRDQPIVKTKISIETRSDNKENTTFGPTREKHLV
LNGGNRNPTGEVVARSAARDYQNSTKKRSSKNLIKGQWTAEEDRKLIRLVRQHGERKWAMISEKLEGRAG
KQCRERWHNHLRPDIKKDGWSEEEERVLVESHMRIGNKWAEIAKLIPGRTENSIKNHWNATKRRQNSKRK
HKRESNADNNDRDASPSAKRPCILQDYIKSIERNNINKDNDEKKNENTISVISTPNLDQIYSDGDSASSI
LGGPYDEELDYFQNIFANHPISLENLGLSQTSDEVTQSSSSGFMIKNPNPNLHDSVGIHHQEATITAPAN
TPHLASDIYLSYLLNGTTSSYSDTHFPSSSSSTSSTTVEHGGHNEFLEPQANSTSERREMDLIEMLSGSI
QGSNICFPLV
>ATMYB120
MIMYGGGGAGKDGGSTNHLSDGGVILKKGPWTAAEDEILAAYVRENGEGNWNAVQKNTGLARCGKSCRL
RWANHLRPNLKKGSFTGDEERLIIQLHAQLGNKWARMAAQLPGRTDNEIKNYWNTRLKRLLRQGLPLYPP
DIIPNHQLHPHPHHQQQQHNHHHHHHQQQQHQQMYFQPQSSQRNTPSSSPLPSPTPANAKSSSSFTFH

TTTANLLHPLSPHTPNTPSQLSSTPPPPPLSSPLCSPRNNQYPTLPLFALPRSQINNNNNGNFTFPRPPPLLQPPS
SLFAKRYNNANTPLNCINRVSTAPFSPVSRDSYTSFLTLPYPSPTAQTATYHNTNNPYSSSPSFSLNPSSSSYPTST
SSPSFLHSHYTPSSTSFHTNPVYSMKQEQLPSNQIPQIDGFNNVNNFTDNERQNHNLNSSGAHRRSSSCSLLE
DVFEEAEALASGGRGRPPKRRQLTASLPNHNNNTNNNDNFFSVSFGHYDSSDNLCSLQGKTKTTYNTSNLN
YSSLQVKCKMFMIKTQI
>ATMYB121
MLDWGVQGHHQKHDHDIYQQQHQQQGCRKGPWTLEEDKLLAEYVTSHGEGRWSTVAKCAGLNRSGKSC
RL
RWVNYLRPGLKRGQITPQEEGIILELHSLWGNKWSTIARYLPGRTDNEIKNYWRTHYKKNQKSSSKQDKV
KKSLSRKQQQVDLKPQPQAQSENHQSQLVSQDHMNIDNDHNIASSLYYPTSVFDDKLYMPQSVATTSSDH
SMIDEGHLWGSLWNLDEDDPHSFGGGSGQGTAADIDEKFPDSGIEAPSCGSGDYSYTGVYMGGYIF
>ATMYB122
MVRTPCCRAEGLKKGAWTQEEDQKLIAYVQRHGEGGWRTLPDKAGLKRCGKSCRLRWANYLRPDIKRGEF
SQDEEDSIINLHAIHGNKWSAIARKIPRRTDNEIKNHWNTHIKKCLVKKGIDPLTHKSLLDGAGKSSDHS
AHPEKSSVHDDKDDQNSNNKKLSGSSSARFLNRVANRFGHRINHNVLSDIIGSNGLLTSHTTPTTSVSEG
ERSTSSSSTHTSSNLPINRSITVDATSLSSSTFSDSPDPCLYEEIVGDIEDMTRFSSRCLSHVLSHEDLL
MSVESCLENTSFMREITMIFQEDKIETTSFNDSYVTPINEVDDSCEGIDNYFG
>ATMYB123
MGKRATTSVRREELNRGAWTDHEDKILRDYITTHGEGKWSTLPNQAGLKRCGKSCRLRWKNYLRPGIKRGN
ISSDEEELIIRLHNLLGNRWSLIAGRLPGRTDNEIKNHWNSNLRKRLPKTQTKQPKRIKHSTNNENNVCVIRT
KAIRCSKTLLFSDLSLQKKSSTSPLPLKEQEMDQGGSSLMGDLEFDFDRIHSEFHFPDLMDFDGLDCGNVTSLV
SSNEILGELVPAQGNLDLNRPFTSCHHRGDDEDWLRDFTC
>ATMYB124
MEDTKKKKKKNINNNQDSKKKERHIVTWSQEEDVILREQITLHGTENWAIIASKFKDKSTRQCRRRWYTYLN
SDFKRGGWSPEEDMLLCEAQRVFGNRWTEIAKVVSGRTDNAVKNRFTTLCKKRAKHEAMTKDSNSNTKRM
LFLDGISTPRKSENETPIAKKLKRSHILDLTEISNYGRAEACVNQQIRSPFSVLARNATGIDSLEEQNQTSNVNE
SDGEGMFLKKDDPKVTALMQQAELLSSLAQKVNADNTEQSMENAWKVLQDFLNKGKENDLFRYGIPDIDF
KIEEFKDLIEDLRSGYEDNQLSWRQPDLHDSPASSEYSSGSTIMVDQSGDKTQPFSADTQTEHKQVGEELLVPK
NPDENMPISGEEKFSSPIQVTPLFRSLADGIPSPQFSESERSFLLKTLGIESSSPCPSANPSKPPPCKRVLLHSL
>ATMYB125
MRKMEAKKEEIKKGPWKAEEDEVLINHVKRYGPRDWSSIRSKGLLQRTGKSCRLRWVNKLRPNLKNGCKFS
ADEERTVIELQSEFGNKWARIATYLPGRTDNDVKNFWSSRQKRLARILHNSSDASSSSFNPKSSSSHRLKGKN
VKPIRQSSQGFGLVEEEVTVSSSCSQMVPYSSDQVGDEVLRLPDLGVKLEHQPFAFGTDLVLAEYSDSQNDAN
QQAISPFSPESRELLARLDDPFYYDILGPADSSEPLFALPQPFFEPSPVPRRCRHVSKDEEADVFLDDFPADMFD
QVDPIPSP
>ATMYB3R1
MKREMKAPTTPLESLQGDLKGKQGRTSGPARRSTKGQWTPEEDEVLCKAVERFQGKNWKKIAECFKDRTD
VQCLHRWQKVLNPELVKGPWSKEEDNTIIDLVEKYGPKKWSTISQHLPGRIGKQCRERWHNHLNPGINKNA
WTQEEELTLIRAHQIYGNKWAELMKFLPGRSDNSIKNHWNSSVKKKLDSYYASGLLDQCQSSPLIALQNKSI
ASSSSWMHSNGDEGSSRPGVDAEESECSQASTVFSQSTNDLQDEVQRGNEEYYMPEFHSGTEQQISNAASHA
EPYYPSFKDVKIVVPEISCETECSKKFQNLNCSHELRTTTATEDQLPGVSNDAKQDRGLELLTHNMDNGGKN
QALQQDFQSSVRLSDQPFLSNSDTDPEAQTLITDEECCRVLFPDNMKDSSTSSGEQGRNMVDPQNGKGSLCS
QAAETHAHETGKVPALPWHPSSSEGLAGHNCVPLLDSDLKDSLLPRNDSNAPIQGCRLFGATELECKTDTN
DGFIDTYGHVTSHGNDDNGGFPEQQGLSYIPKDSLKLVPLNSFSSPSRVNKIYFPIDDKPAEKDKGALCYEPPR
FPSADIPFFSCDLVPSNSDLRQEYSPFGIRQLMISSMNCTTPLRLWDSPCHDRSPDVMLNDTAKSFSGAPSILKK
RHRDLLSPVLDRRKDKKLKRAATSSLANDFSRLDVMLDEGDDCMTSRPSESPEDKNICASPSIARDNRNCAS
ARLYQEMIPIDEEPKETLESGGVTSMQNENGCNDGGASAKNDQETSGSFFELRLCSPGMTRARPDNKVNAS
AKDLSNQHKISLGDFPTEEMSSEPLCTVDSIPLSAIDKTNTAETSFDIENFNIFDGTPFRKLLDTPSPWKSPLLFG
SFLQSPKLPPEITFEDIGCFMSPGERSYDAIGLMKHLSEHSATAYADALEVLGNDTPESILKKRQLNKSIQGKEN
QHQPHDQLGNRSQVECRALDFSDCGTPGKAKVPSASPGGYSSPSSYLLKSCR
>ATMYB3R2
MTESIDLNRSESESDNNTDDVTPIFAIDDSSKGRVSGPTRRSTKGGWTAEEDQILTNVVKKYQGRNWKRIAEC
LPGSEENRRNDVQCQHRWLKVLDPSLQKGAWKKEEDELLSELVKDYMENDRPPWSKISKELPGRIGKQCRE
RWHNHLNPTIIKSPWTREEELILVQAQRGNGNKWAEIAKLLPGRTENNIKNHWNCSVKKRLEQFPSNLFSG
VVYGSKPSSGFEYNFFNQRNTMVESCITSQIKEAAKSPQRDFLDLTLGLNWRSISSTSSLRGEESVSSSVDSVC
ARLNACLETPQNSNNDTVCVKEVREMKERLRMAARTFDTPSIISKTSSPASGLKRLRQKYDTPFPTDARSHMS
SEEDHSVSASPSSKYRFVKRNTCSGSKPLERRLDFDFLLWDEHGRRNGIVNFSVRILPQKSDLKSGLVRPFWLR
>ATMYB3R3

MSSTFNPAASSPDEEGTGEVKIEDQCVENKQSTPASCSSVSEGSAGSSHKSPTIASPATVSPTHRYLGRT
SGPIRRAKGGWTPEEDETLRQAVDTFKGKSWKNIAKSFPDRTEVQCLHRWQKVLNPDLIKGPWTHEEDEK
IVELVEKYGPAKWSIIAQSLPGRIGKQCRERWHNHLNPDINKDAWTTEEEVALMNAHRSHGNKWAEIAKV
LPGRTDNAIKNHWNSSLKKKSEFYLLTGRLPPPTTTRNGVPDSVTKRSSSAQKRVFGSVAQTSSVTTDVN
NLAEDGNGQINSSVPVEEVVAASRMTSLNEYARSPQLPNPEPLPENGGAANNGYHLYYTPQIDYYRASEV
DTQRMYGNECGCSPSASPVSFFTPPPCRNVHSNGSTPRSPESYLREAGRTYPNTPSIFRKRRPRVVVQDN
NNAKKTDEAKEVDQKVNDGKDSSEIQNNGSNAYNLSPPYRIRSKRTAVFKSRQLEFISREEEKADDETKS
SEKDMLIDGDSQLLG
>ATMYB3R4
MEAESSTPQERIPKLRHGRTSGPARRSTRGQWTAEEDEILRKAVHSFKGKNWKKIAEYFKDRTDVQCLHR
WQKVLNPELVKGPWTKEEDEMIVQLIEKYGPKKWSTIARFLPGRIGKQCRERWHNHLNPAINKEAWTQEE
ELLLIRAHQIYGNRWAELTKFLPGRSDNGIKNHWHSSVKKKLDSYMSSGLLDQYQAMPLAPYERSSTLQS
TFMQSNIDGNGCLNGQAENEIDSRQNSSMVGCSLSARDFQNGTINIGHDFHPCGNSQENEQTAYHSEQFY
YPELEDISVSISEVSYDMEDCSQFPDHNVSTSPSQDYQFDFQELSDISLEMRHNMSEIPMPYTKESKEST
LGAPNSTLNIDVATYTNSANVLTPETECCRVLFPDQESEGHSVSRSLTQEPNEFNQVDRRDPILYSSASD
RQISEATKSPTQSSSSRFTATAASGKGTLRPAPLIISPDKYSKKSSGLICHPFEVEPKCTTNGNGSFICI
GDPSSSTCVDEGTNNSSEEDQSYHVNDPKKLVPVNDFASLAEDRPHSLPKHEPNMTNEQHHEDMGASSSL
GFPSFDLPVFNCDLLQSKNDPLHDYSPLGIRKLLMSTMTCMSPLRLWESPTGKKTLVGAQSILRKRTRDL
LTPLSEKRSDKKLEIDIAASLAKDFSRLDVMFDETENRQSNFGNSTGVIHGDRENHFHILNGDGEEWSGK
PSSLFSHRMPEETMHIRKSLEKVDQICMEANVREKDDSEQDVENVEFFSGILSEHNTGKPVLSTPGQSVT
KAEKAQVSTPRNQLQRTLMATSNKEHHSPSSVCLVINSPSRARNKEGHLVDNGTSNENFSIFCGTPFRRG
LESPSAWKSPFYINSLLPSPRFDTDLTIEDMGYIFSPGERSYESIGVMTQINEHTSAFAAFADAMEVSIS
PTNDDARQKKELDKENNDPLLAERRVLDFNDCESPIKATEEVSSYLLKGCR
>ATMYB3R5
MSLSSNPPVCSPEKEERSEMKIEIQCMENKQPLAASCSSASEGSGCFFLKSPEIATPATVSSFPRRTSGP
MRRAKGGWTPEEDETLRRAVEKYKGKRWKKIAEFFPERTEVQCLHRWQKVLNPELVKGPWTQEEDDKIVE
LVKKYGPAKWSVIAKSLPGRIGKQCRERWHNHLNPGIRKDAWTVEEESALMNSHRMYGNKWAEIAKVLPG
RTDNAIKNHWNSSLKKKLEFYLATGNLPPPASKFIVLKDIADGDRDSKQSSATKPFKDSDSLTQTSSGNT
DSNEVGRDHFDSSSALLEEVAASRRIGVNEYACSPVEYKPQLPNLEPISEEVRINSKAYFERSIQRKVEN
GFGTPKHGNLYYKSPLDYYFPSEADLQHMYGYECGCSPGAASPVSLMTTPCNKDSGLTATRSPESFLREA
ARTFPNTPSIFRKRRKVVLAAKTDAVVVVNGVVKEVDRKEESKDMRKSLLLETTDNCSDDEELGLNGNAF
NLSPPYRLRAKRTAVIKSRQLEFTSEKEKQPDNEIEFTSAKEKQPDNEIKTSEEDKPV
>ATMYB4R1
MNRNSLYEADDDDDDDEEDDIGEDLEDLRRACMVSDVNSDQFASKTGSIEPEGVGGGEIPSDSENEDDFE
MLRTIKSQLASSKDAGRSSGPPMGLSLLSDSESEDDFEMIRSIKSQLSLSMDVSLPPIGLSDDEEDDAFE
TLRAIRRRFSAYKNFDSEGKFMNDSHGKKKQVHNSDNEPSSEILSRSNTCESFPDHGKSVVTVPDSEDVQ
DGHMPAASSSFPEAARAFVDAIRRNRAYQKFLRGKLAEIEATIEQNEKHKKNVRIVKDFQASCKRITKLA
LCQRKDPRVELISTRKSGPCDSSEVIGPCDSFEGNDKKISPLTLGPAENPCVENYRMALEKYPISVKRRK
WSTEENKNLAKGLKQEVQKILLSEAIERSSDLEGATYDIDTINESIGNLEITPEMIRQFLPKINWDSLDI
KDRSAAECEARWMSSEDPLINHGPWTAAEDKNLLRTIEQTSLTDWVDIAVSLGTNRTPFQCLARYQRSLN
PSILKKEWTAEEDDQLRTAVELFGEKDWQSVANVLKGRTGTQCSNRWKKSLRPTRKGTWSLEEDKRVKVA
VTLFGSQNWHKISQFVPGRTQTQCRERWLNCLDPKVNRGKWTEEEDEKLREAIAEHGYSWSKVATNLSCR
TDNQCLRRWKRLYPHQVALLQEARRLQKEASVGNFVDRESERPALVTSPILALPDISLEPEPDSVALKKK
RKAKQKKSDAERQPKRRRKGLKNCSGDVCRQENETVCENEPNNGGEERMLALECHDEIQDNAKEKPKQR
R
KSVAETVCENEPNNGGEERMLALECDNEIQDNAKEKRKRRRKSVAETSNNTTGLKKLTPRRRKISAVVPI
KNQDAPN

>Bra022602 (AtMYB82)
MEKREEGRKTHVKRGLWKPEEDMILRSYIDTHGEGNWADISRRSGLKRGG
KSCRLRWKNYLRPNIKRGGMTPQEQDLIIRMHKLLGNRWSLIAGRLPGRT
DNEVKNYWNTHLNKKSSSRKQNAAESVEATPLVDKPVMSTEVRGSHGGGE
GEEGTTTTWMEETNFFARIESPQPLTAHYPDTLCFDPSFAFTDCFPLL
>Bra004162 (AtMYB90)
MEGSSQGLKKGAWTAEEDNLLRQCIDKYGEGKWHQVPLRAGLNRCRKSCR
LRWLNYLKPSIKRGKLNSDEVDLLMRLHKLLGNRWSLIAGRLPGRTANDV
KNYWNTHLSKKHEPGCKTQMKKRNIPCSYTTPAQKIDVFKPRPRSFTVNS
GCSHNNGMPEADIVPLCLGHNDTNNVSENIITCNKDDDKSELVSHLMDGQ

NRWWESLLDESQDPAALFPETTAIKKGATSAFDVEQLWSLLDGETGT
>Bra039763 (AtMYB75)
MEGSPKGLRKGAWTAEEDSLLRQCIDKYGEGKWHQVPLRAGLNRCRKSCR
LRWLNYLKPSIKKGKLSSDEVDLLLRLHRLLGNRWSLIAGRLPGRTANDV
KNYWNTHLSKKHEPGCNTKMRKRNIPCSSTQPAQKNEVLKPRPRSFTVNN
GCSHFNGQPKVDVIPLFLGVNNTNNVCENSITYKKDAEKYELVNNLMDGE
NMWWKSLLEESQEPVAIVPESTETEKLATSAFDVEQLWNLLDGETVELD
>Bra001917 (AtMYB75)
MEDSSKGLTKGAWTAEEDSLLRRCIDKYGEGKWHQVPLRAGLNRCRKSCR
LRWLNYLKPNIKRGKLSSDEVDLLLRLHKLLGNRWSLIAGRLPGRTANDI
KNYWNTHLSKKHEPCCKTKMKKRNVTFSSTTPAQKIDVFKPRPRLFTVNN
GCSHLHGLPEVDVVPPCLGLNNINNVCENSMTCNKAGEKYELYSNLMDGE
NMWWESLLEESKQPDGLVPKGTATKKGATFAFDVEQLWNMLDGETVELD
>Bra029113 (AtMYB82)
MEKKEEERKSHVRRGLWKPEEDMILRNCVESHGEGNWADISRRSGLKRGG
KSCRLRWKNYLRPNIKRGGMSPQEQDLIIRMHKLLGNRWSLIAGRLPGRT
DNEVKNYWNTHLNKKSTSRKQNATESVEAPTDKTVMSTGVRHSHGEQGEG
EEGTTNWMEETNCLAYDDHIGSPLPLISHYTDTLVFDPCFAFMDCFPLL
